# Machine learning-based prediction model for the efficacy and safety of statins

**DOI:** 10.3389/fphar.2024.1334929

**Published:** 2024-07-29

**Authors:** Yu Xiong, Xiaoyang Liu, Qing Wang, Li Zhao, Xudong Kong, Chunhe Da, Zuohuan Meng, Leilei Qu, Qinfang Xia, Lihong Liu, Pengmei Li

**Affiliations:** ^1^ Institute of Materia Medica, Chinese Academy of Medical Sciences and Peking Union Medical College, Beijing, China; ^2^ Department of Pharmacy, China-Japan Friendship Hospital, Beijing, China; ^3^ Department of Pharmacy Administration, Clinical Pharmacy School of Pharmaceutical Sciences, Peking University, Beijing, China; ^4^ Department of Automation, Tsinghua University, Beijing, China; ^5^ Respiratory Center of the Third Affiliated Hospital of Gansu University of Traditional Chinese Medicine, Baiyin, China; ^6^ Institute of Traditional Chinese Medicine, The Third Affiliated Hospital of Gansu University of Chinese Medicine, Baiyin, China; ^7^ Respiratory and Critical Care Medical Center, Baiyin First People’s Hospital, Baiyin, China; ^8^ Department of Information Center, China-Japan Friendship Hospital, Beijing, China

**Keywords:** statins, machine learning, predictive model, random forest, efficacy, safety

## Abstract

**Objective:**

The appropriate use of statins plays a vital role in reducing the risk of atherosclerotic cardiovascular disease (ASCVD). However, due to changes in diet and lifestyle, there has been a significant increase in the number of individuals with high cholesterol levels. Therefore, it is crucial to ensure the rational use of statins. Adverse reactions associated with statins, including liver enzyme abnormalities and statin-associated muscle symptoms (SAMS), have impacted their widespread utilization. In this study, we aimed to develop a predictive model for statin efficacy and safety based on real-world clinical data using machine learning techniques.

**Methods:**

We employed various data preprocessing techniques, such as improved random forest imputation and Borderline SMOTE oversampling, to handle the dataset. Boruta method was utilized for feature selection, and the dataset was divided into training and testing sets in a 7:3 ratio. Five algorithms, including logistic regression, naive Bayes, decision tree, random forest, and gradient boosting decision tree, were used to construct the predictive models. Ten-fold cross-validation and bootstrapping sampling were performed for internal and external validation. Additionally, SHAP (SHapley Additive exPlanations) was employed for feature interpretability. Ultimately, an accessible web-based platform for predicting statin efficacy and safety was established based on the optimal predictive model.

**Results:**

The random forest algorithm exhibited the best performance among the five algorithms. The predictive models for LDL-C target attainment (AUC = 0.883, Accuracy = 0.868, Precision = 0.858, Recall = 0.863, F1 = 0.860, AUPRC = 0.906, MCC = 0.761), liver enzyme abnormalities (AUC = 0.964, Accuracy = 0.964, Precision = 0.967, Recall = 0.963, F1 = 0.965, AUPRC = 0.978, MCC = 0.938), and muscle pain/Creatine kinase (CK) abnormalities (AUC = 0.981, Accuracy = 0.980, Precision = 0.987, Recall = 0.975, F1 = 0.981, AUPRC = 0.987, MCC = 0.965) demonstrated favorable performance. The most important features of LDL-C target attainment prediction model was cerebral infarction, TG, PLT and HDL. The most important features of liver enzyme abnormalities model was CRP, CK and number of oral medications. Similarly, AST, ALT, PLT and number of oral medications were found to be important features for muscle pain/CK abnormalities. Based on the best-performing predictive model, a user-friendly web application was designed and implemented.

**Conclusion:**

This study presented a machine learning-based predictive model for statin efficacy and safety. The platform developed can assist in guiding statin therapy decisions and optimizing treatment strategies. Further research and application of the model are warranted to improve the utilization of statin therapy.

## 1 Introduction

Atherosclerotic cardiovascular disease (ASCVD) is a condition characterized by arterial inflammation and closely linked to lipid abnormalities, causing morbidity and mortality worldwide, casting its widespread impact on over 500 millon individuals and contributing to an alarming toll of 19 million annual fatalities (Barquera et al., 2015; Vasan et al., 2022). Stroke and ischemic heart disease (IHD) are the leading causes of years of life lost (YLLs) in China, which is the main type of ASCVD (Zhou et al., 2019). Low-density lipoprotein (LDL) is causally associated with ASCVD, and reducing LDL cholesterol (LDL-C) can significantly decrease the risk of ASCVD (Li et al., 2021). However, recent studies have indicated a changing global epidemiological profile of lipid level. While elevated cholesterol levels have long been regarded as emblematic of affluent Western nations, the determinants of blood cholesterol—both dietary and behavioral—are undergoing rapid transformations across the globe. Remarkably, over the past decade, China has witnessed an unprecedented surge in LDL-C levels, exemplifying an alarming trajectory (Repositioning of the global epicentre, 2020). The extent of lipid-lowering drug utilization varies greatly among nations, and these fluctuations are highly likely to have had a substantial impact on cholesterol levels in the past decade. In addition, both the US Preventive Services Task Force (USPSTF) and American Heart Association American College of Cardiology (AHA/ACC) guidelines recommend initiating statin therapy for patients aged 40–75 years with elevated 10-year ASCVD risk (Nasir et al., 2015; Mortensen et al., 2016; Zeitouni et al., 2020; Greenland and Lloyd-Jones, 2022; Stone et al., 2022). Therefore, the rational use of statin therapy is crucial. Statins are generally well-tolerated, however, during clinical application, they have been associated with muscle pain, liver enzyme abnormalities, hyperglycemia, and neurological disorders, collectively known as statin-associated symptoms (SAS). Among them, statin-associated muscle symptoms (SAMS) are the most common side effect of statins. It has been reported that SAMS occurs in 10%–25% of patients receiving statin treatment (Thompson et al., 2016). The impact of statins on liver function is dose-dependent, and approximately 1%–3% of patients may experience elevated liver enzyme levels (Farmer and Torre-Amione, 2000). Due to genetic polymorphisms, Chinese individuals have been found to have lower tolerability to statins compared to Caucasians. It has been observed that Chinese patients are more prone to experiencing adverse reactions at the same dosage (Liao, 2007). So achieving optimal efficacy, characterized by reaching guideline-recommended LDL-C levels, while maintaining effective control of adverse reactions is of paramount importance in statin therapy.

With the advancement of scientific and technological innovations, the “medicine+” paradigm has gained prominence, integrating medical big data mining and machine learning techniques into clinical decision-making. These approaches have been widely utilized to optimize treatment and management strategies in various clinical domains, including risk prediction models (Deo, 2015), disease diagnosis (Zhang et al., 2016), and medical image recognition (Shehab et al., 2022). Currently, machine learning methods have been employed to establish predictive models for various blood lipid disorders, including models for predicting familial hypercholesterolemia gene mutations (Besseling et al., 2017) and Traditional Chinese Medicine (TCM) based classification of lipid abnormalities (Liu J. et al., 2023). However, there remains a paucity of prediction models specifically focused on statin therapy. Liu H. et al. (2023) recently developed a prediction model to assess the attainment of LDL-C targets after 1 month of atorvastatin treatment, but this study focused solely on efficacy prediction and did not consider safety outcomes. Additionally, the sample size used in the study was relatively small.

So this study aimed to construct prediction models for the efficacy and safety of statin therapy based on real-world clinical data. The efficacy prediction model focused on predicting LDL-C target attainment (Referred to as Model 1), while the safety prediction models were further divided into sub-models for predicting liver enzyme abnormalities (Referred to as Model 2) and muscle pain or Creatine kinase (CK) abnormalities (Referred to as Model 3). In total, three prediction models were established to provide a practical monitoring tool for the rational application of statins in clinical practice.

## 2 Materials and methods

### 2.1 Data source and collection

We obtained data on all hospitalized patients who had used statins from the Hospital Information System (HIS) of the China-Japan Friendship Hospital and Baiyin First People’s Hospital. The data retrieval period for China-Japan Friendship Hospital was from May 2018 to February 2024, and for Baiyin First People’s Hospital, it was from September 2017 to October 2022. Identifying information such as names, phone numbers, and home addresses will be anonymized to ensure patient confidentiality. And this study has been approved by the Medical Ethics Committee of China-Japan Friendship Hospital and Baiyin First People’s Hospital.

Inclusion criteria: (1) Data of all hospitalized patients who had used statins (including Atorvastatin, Rosuvastatin, Pitavastatin, Simvastatin, Fluvastatin, Pravastatin, Amlodipine and Atorvastatin).

Exclusion criteria: (1) Exclusion of data without dosage of statins; (2) For Model 1, data with empty LDL-C fields were excluded; for Model 2, data with empty alanine aminotransferase (ALT) or aspartate aminotransferase (AST) fields were excluded, and data with diagnoses of “liver disease” or “hepatitis” were also excluded; for Model 3, data with empty CK fields were excluded, and data with diagnoses of “acute myocardial infarction”, “myocarditis”, or “dermatomyositis” were also excluded.

### 2.2 Diagnostic criteria and grouping basis

The criteria for determining LDL-C target attainment are based on the “Guidelines for the Prevention and Treatment of Dyslipidemia in Chinese Adults (Revised in 2016)”: LDL-C < 1.8 mmol/L for extremely high-risk individuals, LDL <2.6 mmol/L for high-risk individuals, and LDL <3.4 mmol/L for moderate and low-risk individuals. A value of 1 is assigned for attainment, while a value of 0 is assigned for non-attainment (specific criteria can be found in the Supplementary Table S1). The criteria for determining liver enzyme abnormalities are as follows: AST or ALT >40 U/L is assigned a value of 1, and *vice versa* was assigned a value of 0. The criteria for determining muscle pain or CK abnormalities are as follows: explicit indication of muscle pain in the medical records after statin use and CK > 200 U/L in laboratory tests are assigned a value of 1, and *vice versa* was assigned a value of 0.

### 2.3 Study variables

There were 47 variables: (1) basic data, including age, sex, Body Mass Index(BMI), smoking history, drink history, length of stay (days); (2) disease data, including number of diseases, Type 2 Diabetes Mellitus (T2DM), hypertension, Coronary Heart Disease (CHD), hypothyroidism, heart failure, atrial fibrillation, depression, epilepsy, chronic liver disease, chronic kidney disease (CKD), Myocardial Infarction (MI) or old MI, stroke, cerebral infarction, statin intolerance, coronary artery stent implantation; (3) drug use information, including number of oral medicines, dose of statins (Principles for the treatment of statin dose are provided in the Supplementary Table S2), amlodipine, ezetimibe, evolocumab, fibrates, niacin, aspirin, clopidogrel, Traditional Chinese Medicine (TCM), cyclosporine, colchicine; (4) laboratory examination, including C-reactive protein (CRP), High density lipoprotein (HDL), Triglyceride (TG), Uric acid (UA), Platelets (PLT), Homocysteine (HCY), ALT, AST, Creatinine (Crea), Systolic blood pressure (SBP), Low density lipoprotein (LDL), Total cholesterol (TC), Creatine kinase (CK).

### 2.4 Data preprocessing

#### 2.4.1 Data pre-screening

(1) Delete columns with a missing data ratio of 90%; (2) Delete columns with a single category ratio of 90%; (3) Delete columns with a coefficient of variation less than 0.1.

#### 2.4.2 Data filling

Two main methods were used. (1) No filling: delete the missing columns in the data in turn, and then delete the missing rows, and finally get the data without missing values. (2) Improved random forest filling: firstly, the missing data is initially filled by linear interpolation; then, the original missing data is combined with the initial filling data into a matrix; finally, a random forest filling model is built to fill the missing data. (Deng et al., 2019).

#### 2.4.3 Data balance

If there is a significant imbalance in the number of positive and negative samples, defined as a difference greater than 2 times, it is considered as an imbalanced dataset. Imbalanced datasets can cause biased model performance, which tends to be biased towards the majority class because it has more instances to learn from. And the model may not accurately capture the underlying patterns in the minority class, affecting its ability to make reliable predictions. Therefore, when the data filling is complete, the data must be checked for imbalance and if there is imbalance, the data must be sampled so that the positive and negative sample sizes of the data for model training are in the correct proportion. Borderline Synthetic Minority Over-sampling Technique (BSMOTE) was used for oversampling, which is a variation of the traditional SMOTE algorithm. Instead of generating synthetic samples indiscriminately for the minority class, BSMOTE focuses on instances near the decision boundary between the minority and majority classes. This helps in creating synthetic samples where they are most needed to improve model generalization. In addition, the implementation of BSMOTE is relatively straightforward, making it accessible for medical practitioner without requiring intricate adjustments (Gao et al., 2020; Han et al., 2005).

The basic flow of the algorithm is:

(1) Find *K* samples of the nearest neighbor for each sample x_i_, whose label is “1”;
(2) A sample x_j_ belonging with few categories is selected randomly from *K*;
(3) Linearly interpolate randomly between x_i_ and x_j_ to construct a new minority sample.

#### 2.4.4 Feature selection

Feature selection is an important aspect of model construction, as it helps eliminate the limitations of relevant variables and unnecessary noise, making the final analysis results closer to reality. In this study, the feature selection method used is Boruta screening, which is based on data sampling and conducted after data imputation.

### 2.5 Model training

During the model training phase, five machine learning algorithms were employed, including logistic regression, naive Bayes, decision tree (DT), random forest (RF), and gradient boosting decision tree (GBDT). These algorithms are chosen because of their respective features and strengths to cope with different types of data and problems. Logistic regression is suitable for linearly divisible problems, DT and RF are able to capture nonlinear relationships and handle high dimensional data, GBDT has high predictive performance, and naive Bayes performs well with large scale data and missing data. By comparing the performance of these algorithms, the most suitable model for the problem at hand can be selected.

Seven evaluation metrics, including area under the receiver operating characteristic curve (AUC), Accuracy, Precision, Recall, F1 Score, Area Under the Precision-Recall Curve (AUPRC) and Matthews correlation coefficient (MCC) were used to assess the performance of the machine learning models. The modeling process was conducted as follows:

(1) The data was divided into a training set and a testing set in a 7:3 ratio.
(2) The training set was fed into the machine learning models, and a ten-fold cross-validation method was applied to adjust the model parameters, aiming to maximize the AUC value on the training set.
(3) The best model parameters obtained from the training set were used to test the models on the testing set. The AUC values obtained from the testing set were used to differentiate the performance of the various machine learning models.

### 2.6 Model validation

Model validation was conducted through both internal and external validation. Hypothesis testing was employed to examine the impact of different data processing methods and algorithms on the predictive performance of the models. Internal validation was performed using ten-fold cross-validation, and external validation was conducted by utilizing bootstrapping sampling and analyzing the results on an independent validation dataset. Meanwhile, in the external validation, another copy of the data, which is completely separated from the training data (training and validation sets), serves as a test set to evaluate the performance of the final model again. The mean ± standard deviation and 95% confidence intervals (CI) were calculated for the seven evaluation metrics (AUC, Accuracy, Precision, Recall, F1 Score, AUPRC and MCC).

Decision curve analysis (DCA) was used to access the model performance. Additionally, sample size validation was carried out to assess the correlation between sample size and model predictive performance. Furthermore, model interpretation analysis based on SHAP (Shapley Additive Explanations) was also conducted.

### 2.7 Model application

Based on the developed optimal prediction models, a web-based platform for predicting the effectiveness and safety of statins was built using the Flask framework. This platform serves as a practical tool for the application and refinement of the models in the future.

### 2.8 Statistical analysis

Categorical variables were presented as percentages and counts, while continuous variables were reported as mean ± standard deviation (SD). Analysis of variance (ANOVA) and rank sum tests were used for univariate analysis. Statistical analysis was performed using the “stats” module in Python 3.8, and model development utilized the “sklearn” library in Python 3.8.

## 3 Results

### 3.1 Description of variables

Model 1 included 3633 samples, with 1153 patients (32%) reaching the target and 2480 patients (68%) not reaching it. A total of 44 variables were considered. For Model 2, 4159 samples were analyzed, with 768 patients (18%) showing abnormal enzyme levels and 3391 patients (82%) having normal levels. A total of 45 variables were included. Regarding Model 3, the sample size was 3345, with 209 patients (6%) experiencing abnormal CK levels and 94% of patients having normal CK levels. A total of 47 variables were analyzed in the study. The information for each variable is presented in Table 1. The results of the ANOVA of the variables in the three models were presented in the Supplementary Tables S3–S5.

**TABLE 1:** Basic characteristics of each variable.

Variables	Parameters	Model 1	Model 2	Model 3
Y1: LDL-C target attainment	N	3633		
	No, n (%)	2480 (0.68)		
	Yes, n (%)	1153 (0.32)		
Y2: Liver enzyme abnormality	N		4159	
	No, n (%)		3391 (0.82)	
	Yes, n (%)		768 (0.18)	
Y3: Muscle pain/CK abnormality	N			3345
	No, n (%)			3136 (0.94)
	Yes, n (%)			209 (0.06)
Age	N	3633	4159	3345
	Mean (SD)	71.09 (11.02)	70.92 (11.01)	71.25 (10.64)
Sex	N	3633	4159	3345
	Male, n (%)	2113 (0.58)	2427 (0.58)	1930 (0.58)
	Female, n (%)	1520 (0.42)	1732 (0.42)	1415 (0.42)
BMI	N	2708	3099	
	Mean (SD)	24.60 (19.56)	24.09 (16.45)	24.42 (15.55)
Smoking history	N	3633	4159	3345
	No, n (%)	1623 (0.45)	2046 (0.49)	1448 (0.43)
	Yes, n (%)	2010 (0.55)	2113 (0.51)	1897 (0.57)
Drink history	N	3633	4159	3345
	No, n (%)	1905 (0.52)	1759 (0.42)	1703 (0.51)
	Yes, n (%)	1728 (0.48)	2400 (0.58)	1642 (0.49)
Length of stay (days)	N	2616	2837	2456
	Mean (SD)	12.21 (13.08)	9.52 (14.00)	12.04 (13.02)
Number of diseases	N	3633	4159	3345
	Mean (SD)	10.40 (7.00)	10.84 (7.23)	10.10 (6.69)
T2DM	N	3633	4159	3345
	No, n (%)	1858 (0.51)	2149 (0.52)	1770 (0.53)
	Yes, n (%)	1775 (0.49)	2010 (0.48)	1575 (0.47)
Hypertension	N	3633	4159	3345
	No, n (%)	1265 (0.35)	1418 (0.34)	1195 (0.36)
	Yes, n (%)	2368 (0.65)	2741 (0.66)	2150 (0.64)
CHD	N	3633	4159	3345
	No, n (%)	2257 (0.62)	2531 (0.61)	2156 (0.64)
	Yes, n (%)	1376 (0.38)	1628 (0.39)	1189 (0.36)
Hypothyroidism	N	3633	4159	3345
	No, n (%)	3487 (0.96)	3995 (0.96)	3223 (0.96)
	Yes, n (%)	146 (0.04)	164 (0.04)	122 (0.04)
Heart failure	N	3633	4159	3345
	No, n (%)	3357 (0.92)	3810 (0.92)	3156 (0.94)
	Yes, n (%)	276 (0.08)	349 (0.08)	189 (0.06)
Atrial fibrillation	N	3633	4159	3345
	No, n (%)	3261 (0.90)	3704 (0.89)	3030 (0.91)
	Yes, n (%)	372 (0.10)	455 (0.11)	315 (0.09)
Depression	N	3633	4159	3345
	No, n (%)	3519 (0.97)	4032 (0.97)	3255 (0.97)
	Yes, n (%)	114 (0.03)	127 (0.03)	90 (0.03)
Epilepsy	N	3633	4159	3345
	No, n (%)	3597 (0.99)	4118 (0.99)	3311 (0.99)
	Yes, n (%)	36 (0.01)	41 (0.01)	34 (0.01)
Chronic liver disease	N	3633	-	3345
	No, n (%)	3578 (0.98)	-	3289 (0.98)
	Yes, n (%)	55 (0.02)	-	56 (0.02)
CKD	N	3633	4159	3345
	No, n (%)	3082 (0.85)	3519 (0.85)	2901 (0.87)
	Yes, n (%)	551 (0.15)	640 (0.15)	444 (0.13)
MI/old MI^a^	N	3633	4159	3345
	No, n (%)	3334 (0.92)	3781 (0.91)	3220 (0.96)
	Yes, n (%)	299 (0.08)	378 (0.09)	125 (0.04)
Stroke	N	3633	4159	3345
	No, n (%)	3610 (0.99)	4133 (0.99)	3329 (0.99)
	Yes, n (%)	23 (0.01)	26 (0.01)	16 (0.01)
Cerebral infarction	N	3633	4159	3345
	No, n (%)	2215 (0.61)	2578 (0.62)	2038 (0.61)
	Yes, n (%)	1418 (0.39)	1581 (0.38)	1307 (0.39)
Statin intolerance	N	3633	4159	3345
	No, n (%)	3615 (0.99)	4134 (0.99)	3335 (0.997)
	Yes, n (%)	18 (0.01)	25 (0.01)	10 (0.003)
Coronary artery stent implantation	N	3633	4159	3345
	No, n (%)	3193 (0.88)	3616 (0.87)	2978 (0.89)
	Yes, n (%)	440 (0.12)	543 (0.13)	367 (0.11)
Number of oral medicines	N	3633	4151	3344
	Mean (SD)	10.10 (5.48)	9.72 (5.34)	9.34 (5.17)
Dose of statins	N	3633	4159	3345
	High dose (%)	205 (0.05)	224 (0.05)	156 (0.04)
	Conventional dose (%)	3427 (0.94)	3934 (0.94)	3188 (0.95)
	Low dose (%)	1 (0.01)	1 (0.01)	1 (0.01)
Amlodipine	N	3633	4159	3345
	No, n (%)	3246 (0.89)	3713 (0.89)	2991 (0.89)
	Yes, n (%)	387 (0.11)	446 (0.11)	354 (0.11)
Ezetimibe	N	3633	4159	3345
	No, n (%)	3166 (0.87)	3591 (0.86)	2995 (0.90)
	Yes, n (%)	467 (0.13)	568 (0.14)	350 (0.10)
Evolocumab	N	3633	4159	3345
	No, n (%)	3547 (0.98)	4057 (0.98)	3288 (0.98)
	Yes, n (%)	86 (0.02)	102 (0.02)	57 (0.02)
Fibrates	N	3633	4159	3345
	No, n (%)	3584 (0.99)	4111 (0.99)	3311 (0.99)
	Yes, n (%)	49 (0.01)	48 (0.01)	34 (0.01)
Niacin	N	3633	4159	3345
	No, n (%)	3616 (0.99)	4142 (0.996)	3330 (0.996)
	Yes, n (%)	17 (0.01)	17 (0.004)	15 (0.004)
Aspirin	N	3633	4159	3345
	No, n (%)	1414 (0.39)	1736 (0.42)	1330 (0.40)
	Yes, n (%)	2219 (0.61)	2423 (0.58)	2015 (0.60)
Clopidogrel	N	3633	4159	3345
	No, n (%)	2208 (0.61)	2582 (0.62)	2085 (0.62)
	Yes, n (%)	1425 (0.39)	1577 (0.38)	1260 (0.38)
TCM	N	3633	4159	3345
	No, n (%)	3550 (0.98)	4067 (0.98)	3270 (0.98)
	Yes, n (%)	83 (0.02)	92 (0.02)	75 (0.02)
Cyclosporine	N	3633	4159	3345
	No, n (%)	3599 (0.99)	4127 (0.99)	3328 (0.99)
	Yes, n (%)	34 (0.01)	32 (0.01)	17 (0.01)
Colchicine	N	3633	4159	3345
	No, n (%)	3629 (0.999)	4155 (0.999)	3342 (0.999)
	Yes, n (%)	4 (0.001)	4 (0.001)	3 (0.001)
CRP	N	1722	2234	1790
	Mean (SD)	15.76 (27.81)	17.34 (28.55)	15.19 (27.61)
HDL	N	3633	3757	3185
	Mean (SD)	1.09 (0.32)	1.10 (0.32)	1.10 (0.31)
TG	N	3633	3759	3187
	Mean (SD)	1.56 (0.99)	1.57 (1.00)	1.55 (0.96)
UA	N	3619	4136	3323
	Mean (SD)	350.84 (167.79)	350.29 (167.96)	351.23 (176.52)
PLT	N	3567	4090	3285
	Mean (SD)	202.10 (65.85)	206.32 (67.551)	205.80 (66.17)
HCY	N	2159	2270	2036
	Mean (SD)	14.16 (9.90)	14.28 (10.15)	13.97 (10.29)
AST	N	3606	-	3326
	Mean (SD)	25.35 (47.36)	-	23.22 (29.13)
ALT	N	3605	-	3321
	Mean (SD)	24.60 (38.97)	-	23.48 (28.16)
Crea	N	3619	4139	3324
	Mean (SD)	86.44 (88.00)	89.42 (93.48)	79.98 (71.27)
SBP	N	1860	1945	1760
	Mean (SD)	129.19 (16.96)	128.82 (18.30)	129.17 (16.54)
LDL	N	-	3759	3187
	Mean (SD)	-	2.50 (0.97)	2.52 (0.96)
TC	N	-	3758	3186
	Mean (SD)	-	4.07 (1.26)	4.10 (1.22)
CK	N	-	3481	-
	Mean (SD)	-	133.53 (483.35)	-

^a^ For Model 3, the variable for MI, only includes patients with old MI.

### 3.2 Data pre-screening

(1) Model 1: After pre-screening, a total of 29 variables remained, including X1 age, X2 sex, X3 BMI, X4 smoking history, X5 drink history, X6 length of stay, X7 number of diseases, X8 T2DM, X9 hypertension, X10 CHD, X13 atrial fibrillation, X17 CKD, X20 cerebral infarction, X22 coronary artery stent implantation, X23 number of oral medicines, X25 amlodipine, X26 ezetimibe, X30 aspirin, X31 clopidogrel, X35 CRP, X36 HDL, X37 TG, X38 UA, X39 PLT, X40 HCY, X41 AST, X42 ALT, X43 Crea, X44 SBP.
(2) Model 2: After preprocessing, a total of 30 variables remained, including X1 age, X2 sex, X3 BMI, X4 smoking history, X5 drink history, X6 length of stay, X7 number of diseases, X8 T2DM, X9 hypertension, X10 CHD, X13 atrial fibrillation, X16 CKD, X19 cerebral infarction, X22 coronary artery stent implantation, X23 number of oral medicines, X25 amlodipine, X26 ezetimibe, X31 aspirin, X32 clopidogrel, X36 CRP, X37 HDL, X38 TG, X39 UA, X40 PLT, X41 HCY, X42 Crea, X43 SBP, X44 LDL, X45 TC, X46 CK.
(3) Model 3: After preprocessing, a total of 30 variables remained, including X1 age, X2 sex X3 BMI, X4 smoking history, X5 drink history, X6 length of stay, X7 number of diseases, X8 T2DM, X9 hypertension, X10 CHD, X17 CKD, X20 cerebral infarction, X23 coronary artery stent implantation, X24 number of oral medicines, X26 amlodipine, X27 ezetimibe, X32 aspirin, X33 clopidogrel, X37 CRP, X38 HDL, X39 TG, X40 UA, X41 PLT, X42 HCY, X43 AST, X44 ALT, X45 Crea, X46 SBP, X47 LDL, X48 TC.

### 3.3 Data imputation

(1) Model 1: Without imputation, 845 records remained for analysis. With improved random forest imputation, 2708 records remained for analysis.
(2) Model 2: Without imputation, 826 records remained for analysis, while with improved random forest imputation, 2937 records were included for analysis.
(3) Model 3: Without imputation, 814 records remained for analysis, while with improved random forest imputation, 2906 records were included for analysis.

### 3.4 Data balance

(1) Model 1: When data was processed without imputation, there were 845 samples in the datasets after prescreening. The proportion of patients with and without LDL-C compliance was 23.67% vs. 76.33%, so the distribution was uneven. BSMOTE was employed, resulting in 1290 records for subsequent feature selection. On the other hand, when the data was imputed using the improved random forest method, no sample imbalance was present, eliminating the need for sampling.
(2) Model 2: When data was processed without imputation, there were 826 samples in the datasets after prescreening. The proportion of patients with abnormal and normal liver enzymes was 15.01% vs. 84.99%, so the distribution was uneven. With the improved random forest imputation method, there were 2937 samples in the datasets. The proportion of patients with abnormal and normal liver enzymes was 16.48% vs. 83.52%, so the distribution was also uneven. Therefore, BSMOTE was employed, resulting in 1404 and 4906 records for subsequent feature selection without and with improved random forest imputation, respectively.
(3) Model 3: When data was processed without imputation, there were 804 samples in the datasets after prescreening. The proportion of patients with muscle pain/CK abnormality and no muscle pains/CK normal was 7.00% vs. 93.00%, so the distribution was uneven. With the improved random forest imputation method, there were 2521 samples in the datasets. The proportion of patients with muscle pain/CK abnormality and no muscle pains/CK normal was 6.19% vs. 93.81%, so the distribution was also uneven. Therefore, BSMOTE was employed, resulting in 1514 and 4730 records for subsequent feature selection without and with improved random forest imputation, respectively.

The proportions of positive and negative samples for each model and the results after processing by the balancing algorithm were shown in Supplementary Figure S1.

### 3.5 Feature selection

(1) Model 1: 23 variables selected using Boruta screening with no imputation and BSMOTE up-sampling. And all 29 pre-selected variables were included with improved random forest imputation and no data sampling.
(2) Model 2: 21 variables were selected using non-imputed data with BSMOTE up-sampling, and 30 pre-selected variables were included using improved random forest imputation and BSMOTE up-sampling.
(3) Model 3: 26 variables were selected for model development using non-imputed data with BSMOTE up-sampling, while all 30 pre-selected variables were included when using improved random forest imputation with BSMOTE up-sampling.

### 3.6 Model establishment

**FIGURE 1:**
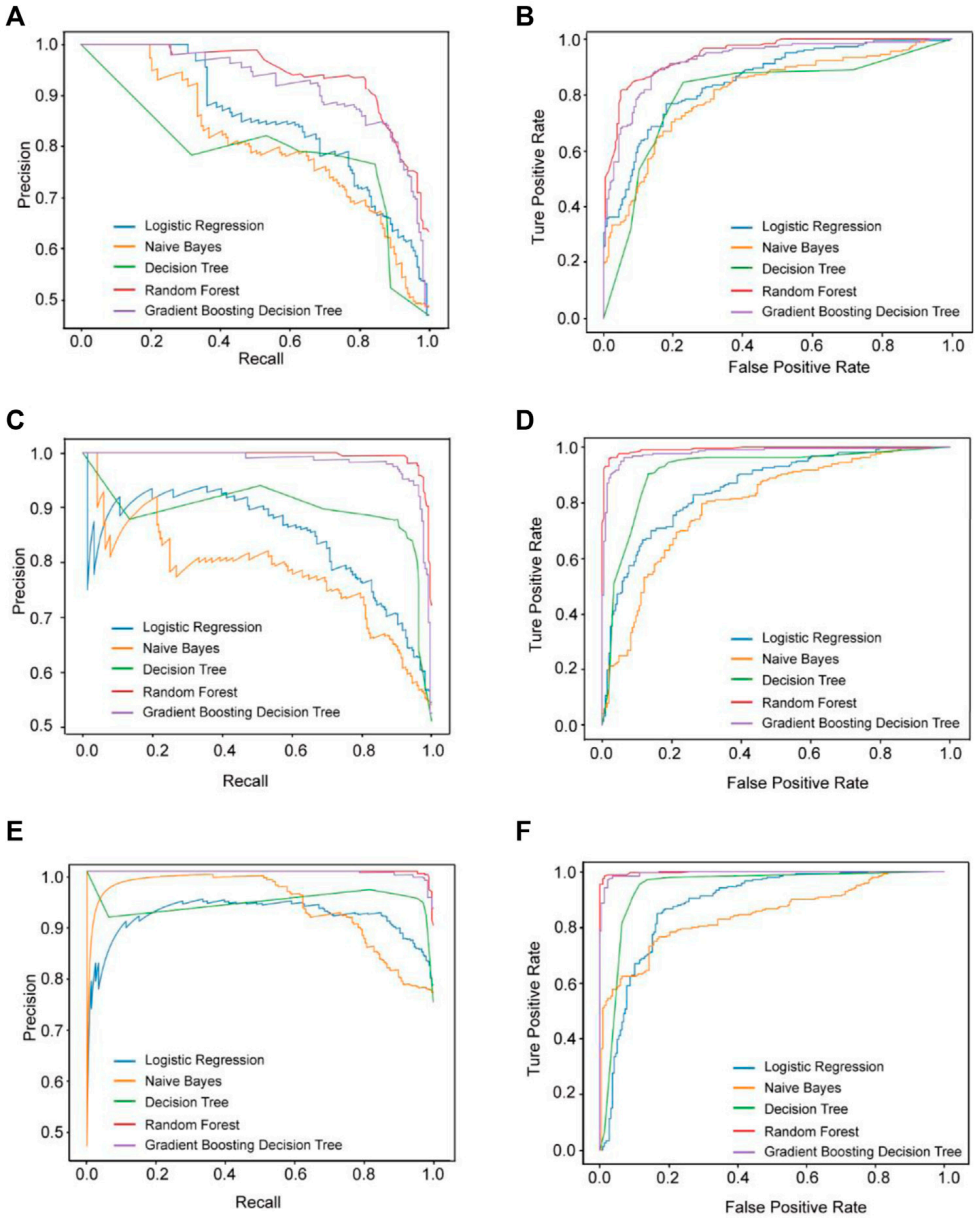
The performance of these three prediction models. The results of AUPRC **(A)** and AUC **(B)** in Model 1. The results of AUPRC **(C)** and AUC **(D)** in Model 2. The results of AUPRC **(E)** and AUC **(F)** in Model 3. AUPRC, area under the precision-recall curve.

During model training, five machine learning algorithms were used to build models based on the data after feature selection. The best-performing models for the three prediction models were all built on non-imputed data with BSMOTE up-sampling as well as RF machine learning methods. The AUC values for these models were 0.883, 0.964, and 0.981, respectively. The MCC values for these models were 0.761, 0.938, and 0.965, respectively. The performance of the models is shown in Figure 1 and Table 2.

**TABLE 2:** The summary of all prediction results in the models.

	Auc	Accuracy	Precision	Recall	F1	AUPRC	MCC
Model 1							
Logistic regression	0.766	0.765	0.733	0.786	0.759	0.810	0.531
Naive bayes	0.746	0.747	0.726	0.742	0.734	0.794	0.492
Decision tree	0.787	0.780	0.787	0.731	0.758	0.828	0.580
Random forest	0.883	0.868	0.858	0.863	0.860	0.906	0.761
GBDT	0.867	0.868	0.843	0.885	0.885	0.889	0.737
Model 2							
Logistic regression	0.772	0.773	0.773	0.787	0.780	0.834	0.544
Naive bayes	0.720	0.718	0.784	0.620	0.693	0.799	0.449
Decision tree	0.880	0.882	0.855	0.926	0.889	0.906	0.756
Random forest	0.964	0.964	0.967	0.963	0.965	0.978	0.938
GBDT	0.941	0.941	0.953	0.931	0.941	0.959	0.882
Model 3							
Logistic regression	0.837	0.837	0.842	0.849	0.846	0.885	0.674
Naive bayes	0.721	0.727	0.699	0.845	0.765	0.813	0.459
Decision tree	0.919	0.921	0.901	0.954	0.927	0.937	0.847
Random forest	0.981	0.980	0.987	0.975	0.981	0.987	0.965
GBDT	0.968	0.969	0.959	0.983	0.971	0.976	0.939

### 3.7 Model validation

**FIGURE 2:**
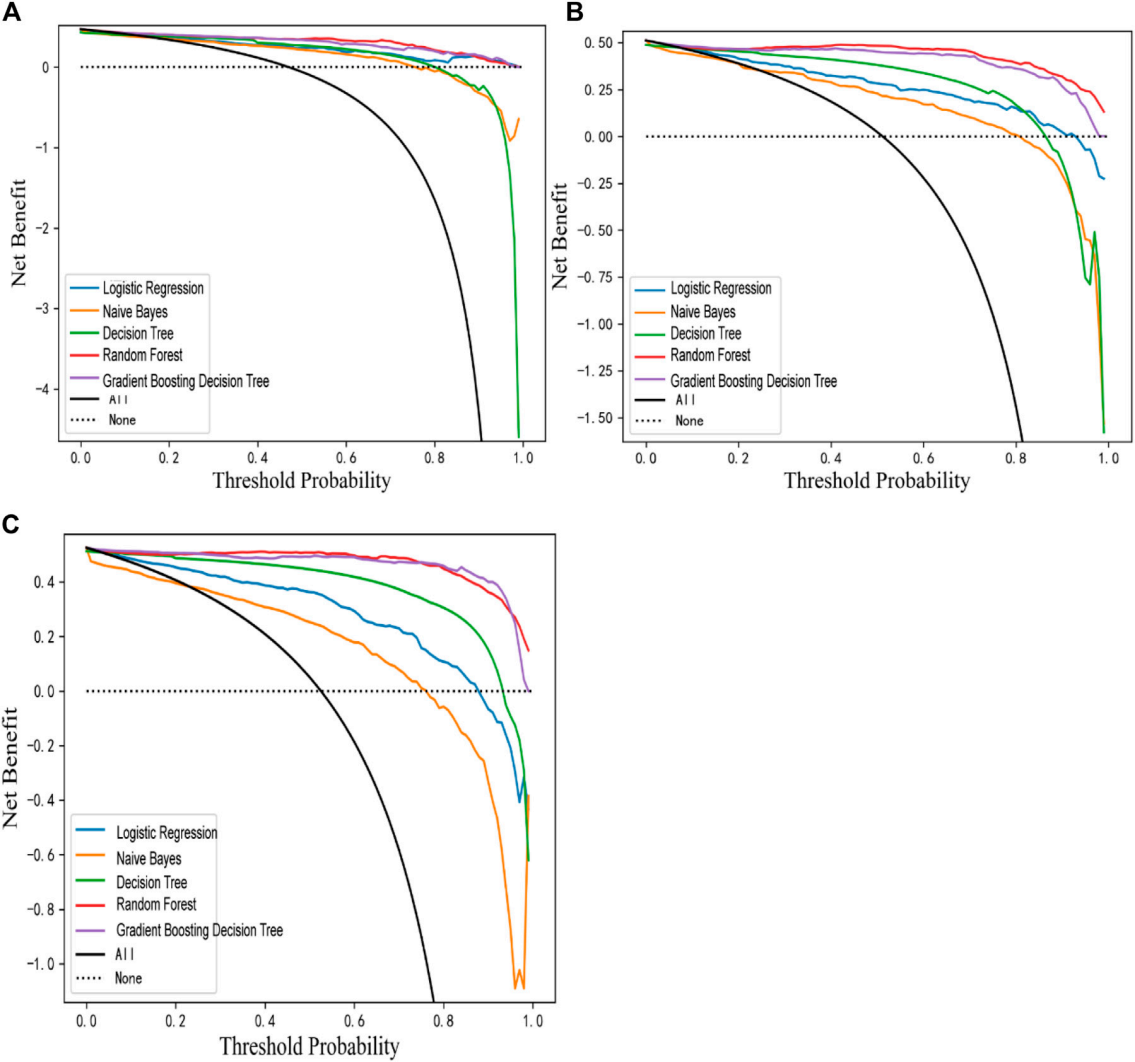
DCA plots of Model 1 **(A)**, Model 2 **(B)** and Model 3 **(C)**.

The three models were internally and externally validated using ten-fold cross-validation and bootstrapping sampling. The mean ± SD and 95% CI of five evaluation metrics (AUC, Accuracy, Precision, Recall, F1 Score, MCC) were calculated. The detailed information is shown in Table 3. And the validation results for the test set were shown in Supplementary Table S6. DCA showed excellent predictive performances (Figure 2).

**TABLE 3:** The results of internal and external validation in the models.

	Auc		Accuracy		Precision		Recall		F1		MCC	
	Mean ± SD	95%CI	Mean ± SD	95%CI	Mean ± SD	95%CI	Mean ± SD	95%CI	Mean ± SD	95%CI	Mean ± SD	95%CI
Model 1												
Internal validation												
Not Imputing												
Logistic regression	0.853 ± 0.030	0.834,0.871	0.775 ± 0.030	0.757,0.794	0.776 ± 0.040	0.751,0.801	0.795 ± 0.042	0.769,0.821	0.784 ± 0.027	0.767,0.800	0.803 ± 0.028	0.785,0.820
Naive bayes	0.798 ± 0.039	0.774,0.822	0.728 ± 0.052	0.695,0.760	0.733 ± 0.062	0.694,0.771	0.752 ± 0.060	0.714,0.789	0.739 ± 0.043	0.713,0.766	0.746 ± 0.038	0.722,0.769
Decision tree	0.781 ± 0.053	0.748,0.814	0.772 ± 0.043	0.745,0.798	0.770 ± 0.060	0.733,0.807	0.803 ± 0.055	0.769,0.837	0.783 ± 0.035	0.762,0.805	0.890 ± 0.035	0.868,0.911
Random forest	0.959 ± 0.011	0.952,0.966	0.881 ± 0.027	0.865,0.898	0.885 ± 0.043	0.858,0.911	0.888 ± 0.038	0.864,0.911	0.885 ± 0.026	0.869,0.901	0.972 ± 0.015	0.962,0.981
GBDT	0.931 ± 0.022	0.918,0.945	0.839 ± 0.038	0.816,0.863	0.833 ± 0.052	0.801,0.866	0.864 ± 0.044	0.837,0.891	0.847 ± 0.034	0.826,0.868	0.957 ± 0.027	0.941,0.974
Improved Random Forest												
Logistic regression	0.749 ± 0.027	0.732,0.766	0.693 ± 0.024	0.678,0.708	0.694 ± 0.025	0.679,0.709	0.699 ± 0.031	0.680,0.718	0.696 ± 0.025	0.680,0.712	0.784 ± 0.027	0.767,0.800
Naive bayes	0.702 ± 0.044	0.674,0.729	0.596 ± 0.032	0.576,0.616	0.562 ± 0.021	0.549,0.575	0.894 ± 0.032	0.875,0.914	0.690 ± 0.022	0.676,0.704	0.739 ± 0.043	0.713,0.766
Decision tree	0.833 ± 0.022	0.820,0.847	0.763 ± 0.024	0.748,0.778	0.788 ± 0.035	0.767,0.810	0.725 ± 0.041	0.700,0.751	0.754 ± 0.027	0.738,0.771	0.776 ± 0.042	0.751,0.802
Random forest	0.946 ± 0.012	0.939,0.954	0.859 ± 0.021	0.846,0.872	0.882 ± 0.041	0.857,0.908	0.834 ± 0.033	0.813,0.855	0.856 ± 0.021	0.844,0.869	0.878 ± 0.030	0.859,0.897
GBDT	0.919 ± 0.008	0.914,0.923	0.834 ± 0.021	0.821,0.848	0.857 ± 0.029	0.840,0.875	0.806 ± 0.039	0.781,0.830	0.830 ± 0.024	0.815,0.845	0.845 ± 0.033	0.824,0.865
External validation												
Not Imputing												
Logistic regression	0.854 ± 0.019	0.853,0.855	0.764 ± 0.022	0.763,0.765	0.733 ± 0.033	0.731,0.734	0.784 ± 0.031	0.783,0.785	0.757 ± 0.025	0.756,0.758	0.758 ± 0.025	0.757,0.759
Naive bayes	0.811 ± 0.022	0.810,0.812	0.747 ± 0.022	0.746,0.748	0.727 ± 0.033	0.725,0.728	0.741 ± 0.033	0.740,0.743	0.733 ± 0.026	0.732,0.735	0.733 ± 0.026	0.731,0.734
Decision tree	0.806 ± 0.024	0.805,0.807	0.781 ± 0.021	0.780,0.782	0.788 ± 0.032	0.786,0.789	0.731 ± 0.033	0.730,0.733	0.758 ± 0.025	0.757,0.759	0.772 ± 0.025	0.771,0.773
Random forest	0.947 ± 0.010	0.947,0.948	0.868 ± 0.017	0.867,0.869	0.858 ± 0.025	0.857,0.859	0.862 ± 0.026	0.861,0.863	0.860 ± 0.019	0.859,0.861	0.869 ± 0.019	0.868,0.870
GBDT	0.925 ± 0.013	0.925,0.926	0.868 ± 0.017	0.867,0.868	0.842 ± 0.026	0.841,0.844	0.884 ± 0.024	0.883,0.885	0.863 ± 0.019	0.862,0.863	0.861 ± 0.019	0.860,0.861
Improved Random Forest												
Logistic regression	0.722 ± 0.015	0.722,0.723	0.668 ± 0.014	0.668,0.669	0.655 ± 0.020	0.654,0.655	0.693 ± 0.019	0.692,0.694	0.673 ± 0.016	0.672,0.674	0.758 ± 0.024	0.757,0.759
Naive bayes	0.668 ± 0.016	0.667,0.669	0.585 ± 0.015	0.584,0.585	0.548 ± 0.016	0.548,0.549	0.895 ± 0.013	0.894,0.895	0.680 ± 0.014	0.679,0.680	0.733 ± 0.026	0.732,0.734
Decision tree	0.820 ± 0.012	0.819,0.820	0.727 ± 0.014	0.727,0.728	0.758 ± 0.019	0.758,0.759	0.656 ± 0.020	0.655,0.657	0.703 ± 0.016	0.703,0.704	0.771 ± 0.025	0.770,0.772
Random forest	0.935 ± 0.007	0.934,0.935	0.843 ± 0.011	0.843,0.844	0.850 ± 0.015	0.849,0.851	0.829 ± 0.016	0.828,0.829	0.839 ± 0.012	0.838,0.839	0.873 ± 0.018	0.872,0.874
GBDT	0.893 ± 0.009	0.893,0.894	0.806 ± 0.012	0.806,0.807	0.824 ± 0.016	0.823,0.825	0.772 ± 0.018	0.771,0.772	0.797 ± 0.013	0.796,0.797	0.863 ± 0.018	0.863,0.864
Model 2												
Internal validation												
Not Imputing												
Logistic regression	0.861 ± 0.043	0.835,0.888	0.812 ± 0.052	0.779,0.844	0.802 ± 0.069	0.759,0.846	0.831 ± 0.037	0.808,0.854	0.815 ± 0.043	0.788,0.842	0.815 ± 0.043	0.788,0.842
Naive bayes	0.806 ± 0.066	0.765,0.847	0.737 ± 0.075	0.691,0.784	0.773 ± 0.089	0.718,0.828	0.669 ± 0.106	0.603,0.735	0.714 ± 0.085	0.661,0.766	0.714 ± 0.085	0.661,0.766
Decision tree	0.886 ± 0.043	0.859,0.913	0.864 ± 0.042	0.838,0.891	0.845 ± 0.061	0.808,0.883	0.895 ± 0.043	0.868,0.922	0.868 ± 0.039	0.844,0.892	0.861 ± 0.031	0.842,0.880
Random forest	0.983 ± 0.013	0.975,0.992	0.952 ± 0.022	0.938,0.966	0.963 ± 0.026	0.947,0.979	0.940 ± 0.049	0.910,0.971	0.951 ± 0.025	0.935,0.966	0.944 ± 0.028	0.926,0.962
GBDT	0.967 ± 0.017	0.956,0.977	0.922 ± 0.034	0.901,0.943	0.924 ± 0.044	0.897,0.951	0.920 ± 0.057	0.884,0.955	0.920 ± 0.037	0.898,0.943	0.919 ± 0.034	0.898,0.940
Improved Random Forest												
Logistic regression	0.725 ± 0.029	0.707,0.743	0.665 ± 0.027	0.649,0.682	0.676 ± 0.034	0.655,0.697	0.648 ± 0.024	0.633,0.663	0.661 ± 0.022	0.648,0.675	0.656 ± 0.031	0.636,0.675
Naive bayes	0.716 ± 0.033	0.696,0.736	0.675 ± 0.028	0.657,0.693	0.697 ± 0.042	0.671,0.722	0.638 ± 0.058	0.602,0.674	0.664 ± 0.032	0.644,0.684	0.661 ± 0.027	0.644,0.678
Decision tree	0.885 ± 0.019	0.874,0.897	0.826 ± 0.020	0.814,0.839	0.855 ± 0.026	0.839,0.871	0.791 ± 0.044	0.764,0.818	0.821 ± 0.024	0.806,0.836	0.828 ± 0.021	0.815,0.841
Random forest	0.978 ± 0.004	0.976,0.981	0.940 ± 0.010	0.934,0.947	0.989 ± 0.008	0.984,0.994	0.891 ± 0.018	0.880,0.903	0.938 ± 0.011	0.931,0.944	0.939 ± 0.012	0.931,0.946
GBDT	0.956 ± 0.011	0.949,0.962	0.912 ± 0.014	0.904,0.921	0.956 ± 0.016	0.947,0.966	0.866 ± 0.025	0.850,0.881	0.909 ± 0.015	0.899,0.918	0.906 ± 0.014	0.897,0.914
External validation												
Not Imputing												
Logistic regression	0.855 ± 0.019	0.854,0.856	0.772 ± 0.021	0.771,0.772	0.771 ± 0.029	[0.770,0.773	0.787 ± 0.028	0.785,0.788	0.779 ± 0.023	0.778,0.780	0.780 ± 0.022	0.779,0.781
Naive bayes	0.796 ± 0.022	0.795,0.797	0.718 ± 0.022	0.717,0.719	0.783 ± 0.031	0.782,0.784	0.621 ± 0.033	0.619,0.622	0.692 ± 0.027	0.691,0.693	0.691 ± 0.026	0.690,0.692
Decision tree	0.915 ± 0.015	0.914,0.916	0.882 ± 0.016	0.881,0.882	0.855 ± 0.023	0.854,0.856	0.926 ± 0.018	0.925,0.927	0.889 ± 0.016	0.888,0.890	0.887 ± 0.016	0.886,0.887
Random forest	0.993 ± 0.003	0.993,0.993	0.964 ± 0.009	0.964,0.965	0.967 ± 0.012	0.967,0.968	0.963 ± 0.013	0.962,0.964	0.965 ± 0.009	0.965,0.966	0.953 ± 0.011	0.953,0.953
GBDT	0.981 ± 0.006	0.981,0.981	0.941 ± 0.011	0.940,0.941	0.953 ± 0.014	0.952,0.954	0.930 ± 0.017	0.930,0.931	0.941 ± 0.011	0.941,0.942	0.942 ± 0.012	0.941,0.942
Improved Random Forest												
Logistic regression	0.755 ± 0.012	0.755,0.756	0.697 ± 0.012	0.697,0.698	0.701 ± 0.017	0.700,0.701	0.669 ± 0.018	0.669,0.670	0.684 ± 0.014	0.684,0.685	0.670 ± 0.014	0.669,0.670
Naive bayes	0.713 ± 0.013	0.712,0.713	0.671 ± 0.012	0.670,0.671	0.700 ± 0.019	0.699,0.701	0.575 ± 0.019	0.574,0.576	0.631 ± 0.016	0.631,0.632	0.675 ± 0.015	0.675,0.676
Decision tree	0.902 ± 0.008	0.902,0.902	0.835 ± 0.009	0.835,0.835	0.869 ± 0.013	0.869,0.870	0.781 ± 0.015	0.780,0.782	0.823 ± 0.011	0.822,0.823	0.814 ± 0.012	0.813,0.815
Random forest	0.979 ± 0.003	0.979,0.979	0.933 ± 0.007	0.933,0.933	0.991 ± 0.004	0.990,0.991	0.871 ± 0.013	0.871,0.872	0.927 ± 0.007	0.927,0.927	0.927 ± 0.007	0.927,0.928
GBDT	0.956 ± 0.005	0.956,0.957	0.898 ± 0.008	0.897,0.898	0.969 ± 0.007	0.969,0.969	0.818 ± 0.014	0.817,0.818	0.887 ± 0.009	0.886,0.887	0.902 ± 0.009	0.901,0.902
Model 3												
Internal validation												
Not Imputing												
Logistic regression	0.880 ± 0.025	0.864,0.895	0.804 ± 0.026	0.788,0.819	0.790 ± 0.031	0.771,0.809	0.818 ± 0.052	0.786,0.851	0.803 ± 0.028	0.785,0.820	0.803 ± 0.028	0.785,0.820
Naive bayes	0.845 ± 0.035	0.823,0.866	0.716 ± 0.047	0.687,0.745	0.665 ± 0.043	0.639,0.692	0.851 ± 0.048	0.822,0.881	0.746 ± 0.038	0.722,0.769	0.746 ± 0.038	0.722,0.769
Decision tree	0.910 ± 0.027	0.893,0.927	0.889 ± 0.038	0.865,0.912	0.868 ± 0.047	0.839,0.897	0.913 ± 0.039	0.889,0.937	0.889 ± 0.037	0.866,0.912	0.890 ± 0.035	0.868,0.911
Random forest	0.994 ± 0.008	0.989,0.999	0.977 ± 0.018	0.966,0.988	0.982 ± 0.014	0.974,0.991	0.971 ± 0.025	0.956,0.986	0.977 ± 0.018	0.965,0.988	0.972 ± 0.015	0.962,0.981
GBDT	0.990 ± 0.009	0.985,0.996	0.956 ± 0.028	0.938,0.973	0.943 ± 0.038	0.920,0.966	0.969 ± 0.023	0.955,0.983	0.956 ± 0.028	0.938,0.973	0.957 ± 0.027	0.941,0.974
Improved Random Forest												
Logistic regression	0.819 ± 0.028	0.802,0.837	0.758 ± 0.015	0.749,0.767	0.757 ± 0.025	0.741,0.772	0.769 ± 0.029	0.751,0.787	0.762 ± 0.013	0.754,0.770	0.770 ± 0.021	0.756,0.783
Naive bayes	0.707 ± 0.028	0.690,0.724	0.633 ± 0.036	0.611,0.656	0.605 ± 0.030	0.587,0.624	0.789 ± 0.044	0.762,0.817	0.685 ± 0.031	0.665,0.704	0.683 ± 0.013	0.675,0.691
Decision tree	0.928 ± 0.025	0.913,0.944	0.895 ± 0.025	0.880,0.911	0.872 ± 0.032	0.851,0.892	0.931 ± 0.026	0.915,0.947	0.900 ± 0.023	0.885,0.914	0.895 ± 0.012	0.888,0.903
Random forest	0.996 ± 0.004	0.994,0.998	0.982 ± 0.007	0.977,0.986	0.995 ± 0.006	0.991,0.999	0.968 ± 0.012	0.961,0.976	0.981 ± 0.007	0.977,0.986	0.982 ± 0.005	0.979,0.985
GBDT	0.990 ± 0.005	0.987,0.994	0.966 ± 0.008	0.961,0.972	0.976 ± 0.010	0.970,0.982	0.957 ± 0.012	0.949,0.964	0.966 ± 0.009	0.961,0.972	0.967 ± 0.006	0.963,0.971
External validation												
Not Imputing												
Logistic regression	0.884 ± 0.017	0.883,0.885	0.838 ± 0.017	0.837,0.838	0.843 ± 0.024	0.842,0.844	0.850 ± 0.023	0.849,0.851	0.846 ± 0.018	0.845,0.847	0.845 ± 0.018	0.845,0.846
Naive bayes	0.844 ± 0.018	0.844,0.845	0.727 ± 0.021	0.727,0.728	0.699 ± 0.027	0.698,0.700	0.844 ± 0.023	0.843,0.845	0.764 ± 0.021	0.764,0.765	0.765 ± 0.021	0.764,0.766
Decision tree	0.942 ± 0.013	0.941,0.942	0.921 ± 0.013	0.921,0.922	0.902 ± 0.019	0.901,0.902	0.954 ± 0.013	0.954,0.955	0.927 ± 0.012	0.926,0.928	0.927 ± 0.012	0.926,0.927
Random forest	0.998 ± 0.001	0.998,0.998	0.980 ± 0.007	0.980,0.980	0.987 ± 0.007	0.987,0.988	0.975 ± 0.010	0.974,0.975	0.981 ± 0.006	0.981,0.981	0.983 ± 0.006	0.983,0.983
GBDT	0.995 ± 0.002	0.995,0.996	0.969 ± 0.008	0.969,0.970	0.960 ± 0.013	0.959,0.960	0.983 ± 0.008	0.983,0.984	0.971 ± 0.008	0.971,0.971	0.971 ± 0.008	0.971,0.971
Improved Random Forest												
Logistic regression	0.816 ± 0.011	0.815,0.816	0.746 ± 0.012	0.745,0.746	0.730 ± 0.017	0.729,0.731	0.764 ± 0.016	0.763,0.765	0.746 ± 0.013	0.746,0.747	0.845 ± 0.018	0.845,0.846
Naive bayes	0.703 ± 0.014	0.702,0.703	0.606 ± 0.013	0.606,0.607	0.569 ± 0.016	0.568,0.570	0.806 ± 0.015	0.805,0.806	0.667 ± 0.013	0.666,0.668	0.765 ± 0.021	0.764,0.766
Decision tree	0.933 ± 0.007	0.933,0.933	0.892 ± 0.008	0.892,0.892	0.855 ± 0.013	0.855,0.856	0.938 ± 0.009	0.938,0.938	0.895 ± 0.008	0.894,0.895	0.927 ± 0.012	0.926,0.927
Random forest	0.996 ± 0.001	0.996,0.997	0.985 ± 0.003	0.985,0.985	1.000 ± 0.000	1.000,1.000	0.970 ± 0.007	0.969,0.970	0.985 ± 0.003	0.984,0.985	0.983 ± 0.006	0.983,0.983
GBDT	0.991 ± 0.002	0.990,0.991	0.967 ± 0.005	0.967,0.967	0.974 ± 0.006	0.973,0.974	0.958 ± 0.008	0.958,0.958	0.966 ± 0.005	0.966,0.966	0.971 ± 0.008	0.971,0.971

### 3.8 Sample size validation

**FIGURE 3:**
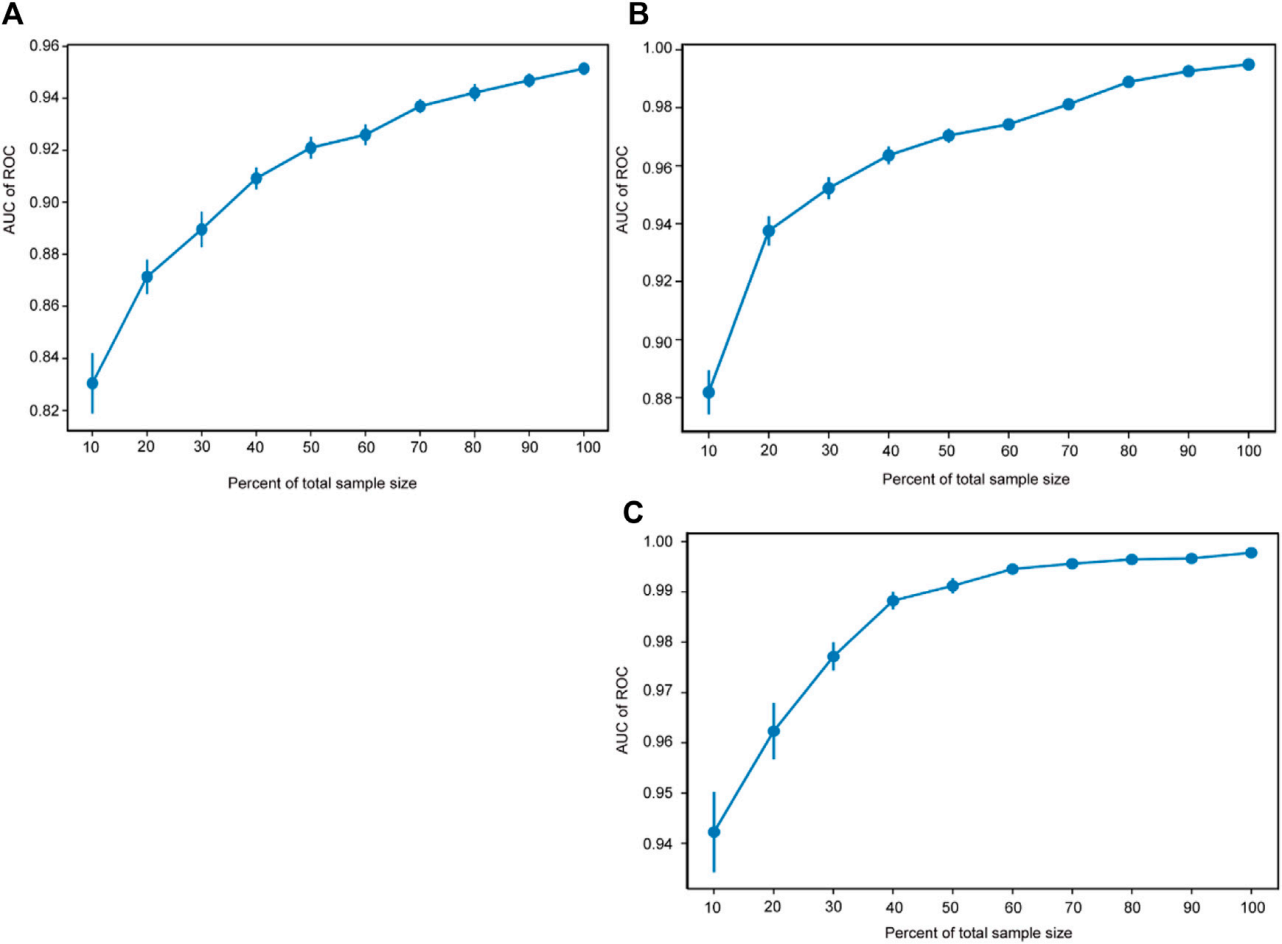
Sample size validation. The sample size validation of Model 1 **(A)**, Model 2 **(B)** and Model 3 **(C)**. ROC, receiver operating characteristic.

The sample size validation was used to assess the correlation between the sample size and the predictive performance of the models. The *X*-axis represents the percentage of the total sample size, while the *Y*-axis represents the AUC values corresponding to different sample sizes. The results of the sample size validation demonstrated that as the sample size increases, the AUC values of the three models gradually rise and then plateau, indicating good predictive performance. This suggests that the sample size used for modeling adequately meets the requirements (shown in Figure 3).

### 3.9 Model interpretability analysis

The study utilized the SHAP model interpretability framework to explain the constructed models. Two interpretation methods were employed: feature density scatter plot and feature importance SHAP values. Feature density scatter plot displays the features sorted by their average absolute SHAP values, indicating their importance to the model. The width of the plot represents sample density, and the color intensity represents the feature value. A dispersed sample distribution indicates a greater feature influence, while a concentration around SHAP = 0 suggests the feature affects only a subset of individuals. And significant features are identified through SHAP values.

**FIGURE 4:**
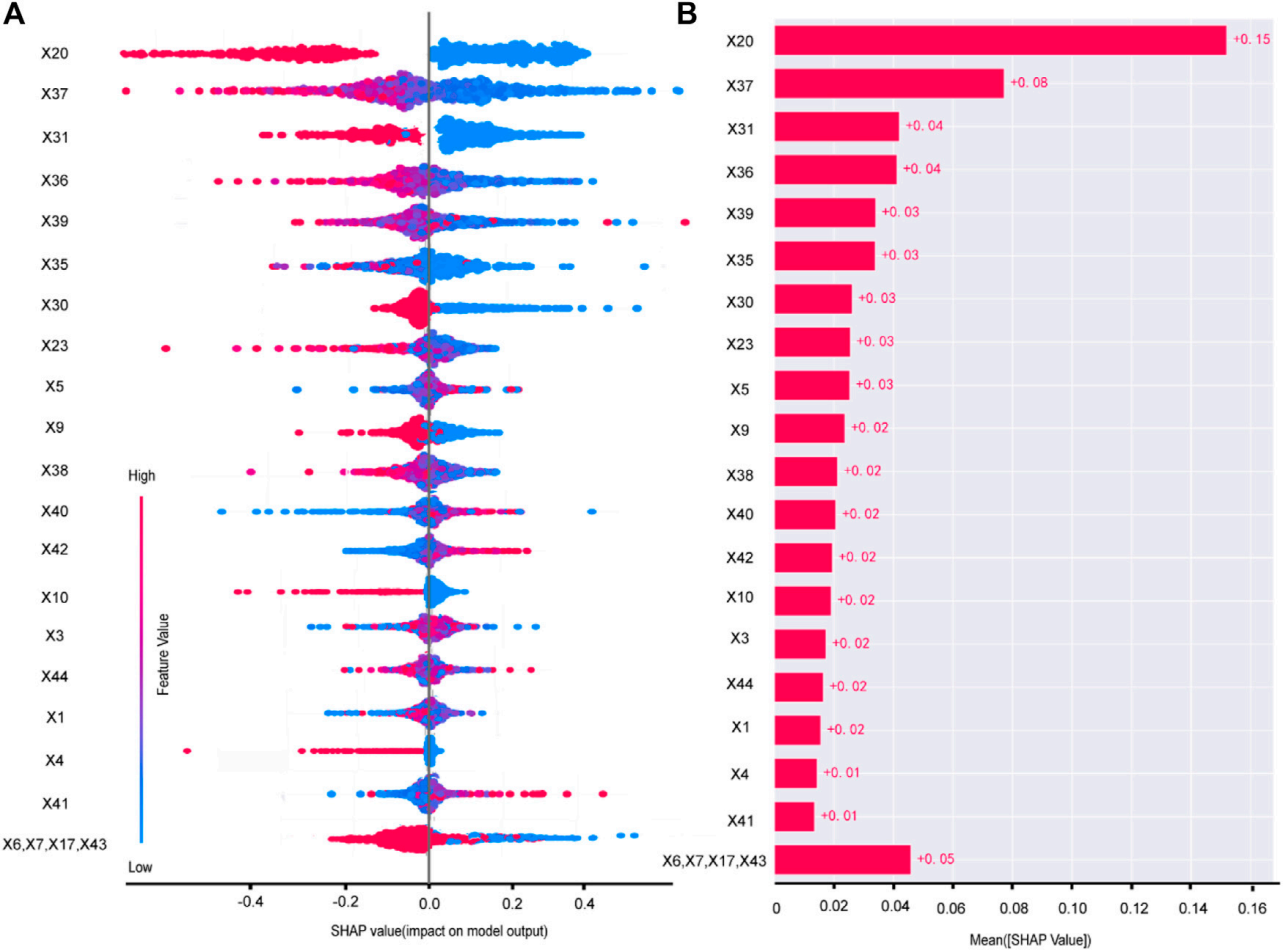
Variable contribution of the Model 1 by SHAP. Summary of SHAP value of each variable **(A)**. Absolute average of SHAP of each variable **(B)**. X1 age, X3 BMI, X4 smoking history, X5 drink history, X6 length of stay, X7 number of diseases, X9 hypertension, X10 CHD, X17 CKD, X20 cerebral infarction, X23 number of oral medicines, X30 aspirin, X31 clopidogrel, X35 CRP, X36 HDL, X37 TG, X38 UA, X39 PLT, X40 HCY, X41 AST, X42 ALT, X43 Crea, X44 SBP.

Taking the example of the LDL-C target attainment prediction model, smaller values of the feature X37-TG will give a positive boost to the predictions of Model 1 (Figure 4A). X20-Cerebral infarction, X37-TG, X31-Clopidogrel and X36-HDL were the top 4 important features (Figure 4B).

The SHAP of Model 2 and Model 3 were shown in Supplementary Figures S2, S3.

### 3.10 Model application

Based on the best-performing model, a web-based platform for predicting the effectiveness and safety of statin drugs was developed using the Flask framework.

**FIGURE 5:**
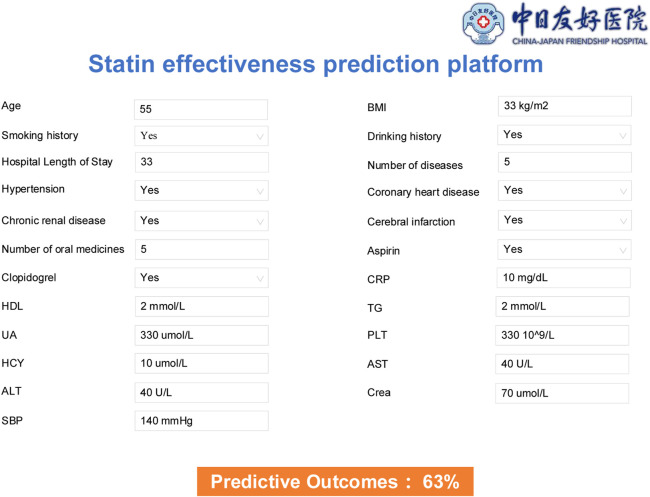
Stain effectiveness prediction platform based on Model 1.

The platform based on the prediction model has been deployed to the Elderly Polypharmacy Risk Alert Platform in China-Japan Friendship Hospital, enabling the application and optimization of the models. By sharing the IP address of the prediction platform to each clinical department in the hospital, especially the cardiovascular department and other key departments, before the use of statins, the prediction model is used to predict the efficacy (LDL-C compliance rate) and safety (incidence of abnormalities in liver enzymes and CK). For patients with high efficacy rate and high risk of adverse reactions, close supervision and monitoring of all relevant indicators are given; for patients with low efficacy rate and high risk of adverse reactions, replacement of lipid-lowering drugs can be considered. Therefore, based on the prediction platform can help clinicians or clinical pharmacists to ensure the lipid-lowering effect while avoiding the occurrence of serious adverse reactions and improving the efficiency of treatment. Model application is the visual display of model training, and the prediction platform built based on model training can be used to solve practical clinical problems in the process of clinical practice, so that the model is not only built, but also can be applied and promoted. The platform of Model 1 was shown in Figure 5. The platform of Model 2 and Model 3 were shown in Supplementary Figures S4, S5.

## 4 Discussion

In this study, we have developed three prediction models for the effectiveness and safety of statins, namely, the LDL target attainment (Model 1), the liver enzyme abnormality (Model 2), and the muscle pain/CK abnormality prediction model (Model 3). Among these models, the RF algorithm has demonstrated the best performance, and AUC value was 0.883, 0.964, and 0.981, respectively.

Of the three prediction models, the best performing models were the unfilled preprocessing method and the RF machine learning algorithm. The better performance of the predictive models obtained by the means of data preprocessing without padding may be due to two reasons, Firstly, the small percentage of missing values, which is less than 1 percent for most of the variables. When the percentage of missing values is relatively low, deleting these variables may not significantly affect the overall model performance, and therefore, computationally populating a small fraction of the missing values may not provide substantial benefits. Secondly, the missing values in this study occurred randomly, and deleting the missing values would maintain the randomness of the data and avoid adversely affecting model training (Ren et al., 2023). RF is an integrated learning method, which means that it combines the predictions of multiple independent models, such as decision trees, to make more accurate and robust predictions. As seen from our study, the RF algorithm consistently outperforms the DT, which shows that the nature of the ensemble helps to reduce overfitting and improves generalization to new, unseen data. In addition, the ability of RF to capture complex non-linear relationships in the data makes it suitable for tasks where the decision boundaries are intricate and cannot be easily represented by a linear model (Hu and Szymczak, 2023). Therefore, the RF algorithm performs better in this study. However, it's essential to note that the performance of machine learning models is highly dependent on the specific characteristics of the dataset, the quality of the features, and the inherent patterns in the data. Different algorithms may excel in different scenarios, and it’s common practice to experiment with multiple algorithms to identify the most suitable one for a given task. Modelling with more data balancing, feature filtering, and machine learning algorithms may produce better performance (Xingwei et al., 2022).

Regarding Model 1, it exhibited good performance (AUC = 0.883, Accuracy = 0.868, Precision = 0.858, Recall = 0.863, F1 = 0.860, AUPRC = 0.906, MCC = 0.761). These results align with a similar model by Liu et al., which evaluated the effectiveness of atorvastatin after 1 month (Liu H. et al., 2023). The RF algorithm also demonstrated superior performance in the studies. However, in Liu et al.’s model, TC was an important variable to predict whether LDL-C would reach the target, resulting in a slightly higher AUC of 0.97 (19). In contrast, our study focused primarily on LDL-C, which is a key component of TC. Thus, TC was not included in our LDL-C prediction model, potentially contributing to the lower AUC. The strong correlation between TC and LDL-C likely contributed to the higher performance observed in Liu et al.’s study. When it comes to the interpretation of the model, the top 5 important feature were cerebral infarction, TG, clopidogrel, HDL and PLT. Consistent with Liu et al.’s study., PLT was found to impact LDL-C target attainment. Kremser et al. also demonstrated a link between PLT hyperactivity and elevated LDL-C, possibly due to the storage and release of pltPCSK9 (platelet-derived proprotein convertase subtilisin/kexin type 9) during PLT activation, which enhances LDL-C levels (Petersen-Uribe et al., 2021). The release of pltPCSK9 may participate in the regulation of plasma LDL-C levels by binding to LDL-C receptors and reducing the uptake of LDL-C particles from the extracellular space into cells, thereby increasing plasma LDL-C concentrations. Similarly, Liu et al. highlighted the importance of TG as a contributing factor, indicating that TG has an impact on LDL-C (Esan and Wierzbicki, 2021; Liu H. et al., 2023). Research suggests that the synthesis of very-low-density lipoprotein (VLDL) is influenced by pathways involving hepatic TG storage (Esan and Wierzbicki, 2021). In addition to TG, HDL has long been one of the key factors in lipid-lowering models (Liu H. et al., 2023) and cardiovascular risk prediction models (Georgoulis et al., 2022). According to the SHAP interpretation, it can be seen that a smaller HDL increases the predictive value of model 1. As to whether higher HDL levels are better for the protection of the cardiovascular system, there is no clear conclusion, and the latest studies have shown that there is a U-shaped relationship between HDL levels and the risk of cardiovascular death, which means that too high or too low a level of HDL is not a good thing (Liu et al., 2022). Cerebral infarction was important factors for LDL-C target achievement because patients with these conditions require strict monitoring of LDL-C levels. Studies have shown that patients with cerebral infarction or transient ischemic attack (TIA) accompanied by atherosclerosis have a lower risk of subsequent cardiovascular events when LDL-C is controlled at lower levels (Amarenco et al., 2020). Therefore, both clinicians and caregivers place greater emphasis on educating and managing patients with these conditions, leading to improved adherence and higher LDL-C target attainment rates.

Regarding the safety assessment of statins, Model 2 exhibited favorable performance (AUC = 0.964, Accuracy = 0.964, Precision = 0.967, Recall = 0.963, F1 = 0.965, AUPRC = 0.978, MCC = 0.938). Similarly, Model 3 also demonstrated excellent performance (AUC = 0.981, Accuracy = 0.980, Precision = 0.987, Recall = 0.975, F1 = 0.981, AUPRC = 0.987, MCC = 0.965). The top 5 important feature of Model 2 were CRP, CK, T2DM, hypertension and the number of oral medications. As for the Model 3, the top 5 important feature were AST, ALT, PLT, clopidogrel and the number of oral medications. In fact, statins have a good safety record in clinical practice, with a liver injury risk of approximately 1%, comparable to placebo. Muscle pain is relatively common, but the rates of myopathy and rhabdomyolysis are low, affecting about 5 and 1.6 individuals per 100,000 people annually, respectively (Gillett and Norrell, 2011). Therefore, there have been no studies conducted to establish predictive models for the safety assessment of statins. So the prediction model established in this study is to provide a practical technical means for the precise administration of statins, to predict the risk of patients before giving statins, and to make a comprehensive judgement in combination with the assessment of effectiveness and safety. If the effectiveness and safety are good, statins are given, and if the effectiveness is poor and safety is poor, statins are not recommended. If effectiveness is good and safety is poor, or if effectiveness is low and safety is good, the dosing regimen needs to be considered in combination with the assessment. In this case, if a decision is made to give statins, a regular monitoring programme needs to be provided, such as regular liver function (AST/ALT) and CK monitoring. Otherwise, switching to other types of lipid-lowering drugs may be considered.

When it comes to the interpretation of the model, in the liver enzyme prediction model, CRP has a significant impact. Regarding the relationship between statins and CRP, previous studies have confirmed that statins can lower CRP levels. However, the extent of CRP reduction varies depending on the specific medication used, similar to the reduction in LDL-C levels (Asher and Houston, 2007; Kandelouei et al., 2022). Even when exploring the rationality of preventive use of statins, CRP and LDL-C are both crucial factors to consider, which indicates a close association between CRP and statins (Dorresteijn et al., 2011). There is limited research on the correlation between CRP and liver enzymes during statin use. However, some studies have reported an association between mild elevation of liver enzymes and higher plasma CRP levels (Kerner et al., 2005). Furthermore, in a comprehensive risk factor study conducted among overweight children and adolescents, both ALT and hs-CRP (high-sensitivity C-reactive protein) were employed as screening indicators for assessing the presence of metabolic syndrome and cardiovascular disease risks (Oliveira et al., 2008). Therefore, further research is needed to determine the correlation between liver enzyme abnormalities and CRP levels following statin use. Also, it is recommended to routinely monitor CRP when using statins (Ridker et al., 2005). In Model 2 and Model 3, ALT or AST and CK were important factors for each other. Liver enzyme elevation and the occurrence of muscle pain with or without an increase in CK are common adverse events during statin use. However, there is limited research exploring the correlation between these factors during the use of statins. A study on patients with rhabdomyolysis observed a significant positive correlation between CK and ALT, AST, Alkaline phosphatase (ALP), and Total bilirubin (TBiL) (Laitselart et al., 2022). Other studies has also explored the clinical and experimental evidence linking elevated transaminases with muscle injury (Lim, 2020). Elevated liver enzymes usually do not directly impact CK levels, but liver dysfunction or disease can occasionally contribute to muscle injury or inflammation, potentially affecting CK levels (Md Sani et al., 2017). Therefore, when using statins, attention should be paid to the possibility of liver enzyme abnormalities and the occurrence of muscle pain or CK abnormalities, although the specific mechanisms are not fully understood. In Model 3, PLT is also one of its important feature. Generally, there is no direct relationship or correlation between CK and PLT due to representing different physiological processes. However, in certain situations, they can independently affect the levels of PLT and CK. For example, in cases of severe liver disease, liver dysfunction can lead to muscle injury, resulting in elevated CK levels. Additionally, liver disease can affect PLT production or function, leading to changes in PLT levels (Wang et al., 2013). Furthermore, the study revealed that the number of oral medications was an important influencing factor for the occurrence of liver enzyme abnormalities and muscle pain or CK abnormalities. Patients with multiple comorbidities often receive multiple drug therapies, which can lead to increased statin exposure due to DDIs and concurrent prescription medications, resulting in an increased risk of adverse events, including SAMA and statin-induced hepatotoxicity (Bellosta and Corsini, 2018).

Currently, articles using machine learning methods to help rationalize the use of statins in the clinic focus more on gene-level research. Many studies have used machine learning combined with genomics (Ooi et al., 2021), transcriptomics (Kim et al., 2014), and also metabolomics (Silveira et al., 2021) to discover relevant SNPs or endogenous metabolites that affect the metabolism of statins, and so on, to explore ways and means to personalize the treatment of statins. Kim et al. used SVM to construct a prediction model and identified 100 signature genes that distinguish between high and low response to statins, providing ideas for identifying new pathways that affect cholesterol metabolism (Kim et al., 2014). Using six machine learning algorithms including logistic regression, elastic nets, RF, boosted trees, neural networks and SVM, Ooi BNS et al. (Ooi et al., 2021) identified groups of SNPs that would be used to predict the incidence of myalgia. Ultimately, a combination of 15 SNPs' was determined to have good predictive performance for predicting myalgia (AUC > 0.9). Silveira AMR et al. (Silveira et al., 2021) used the refined Elastic Net model to look for associations between endogenous metabolites and the corresponding pharmacokinetic parameters, and ultimately identified biomarkers that influence the metabolism of Rosuvastatin (Silveira et al., 2021).

In addition to this, studies relying on electronic health records to construct predictive models for statins are more similar to this study, but there are relatively few such studies. Liu et al. used the RF algorithm to predict the lipid-lowering effect of atorvastatin, which mainly consisted of predicting the levels of LDL, TG, TC, and HDL, with AUCs of 0.97, 0.80, 0.98, and 0.87, respectively (Liu H. et al., 2023). We mainly focused on the effect of lowering LDL, but focused on almost all statins. From the final results, the 23 key features obtained from Model 1 in this study almost included the important features in the study of Liu et al., such as PLT, TG, UA, BMI, and HDL. Although the AUC value (0.883) in this study was lower than that of the LDL prediction model in Liu’s study (0.97), retaining the indicator TC in the LDL prediction model will inevitably lead to a higher performance of the prediction model because LDL is one of the types of TC, and the two variables are highly correlated, which will have an impact on the results. Sun et al. (2023) utilized six machine learning algorithms, including DT, SVM, K-Nearest Neighbours (KNN), RF, and AdaBoost, to construct a prediction model for predicting the occurrence of SAMS with statins (atorvastatin, fluvastatin, lovastatin, pitavastatin, pravastatin, rosuvastatin and simvastatin), and the final model performance was as follows: Precision = 0.85, Recall = 0.71, F1 score = 0.77. Although the optimal algorithm of Sun et al. is a combined rule-based (CRB) algorithm, the screened key variables affecting SAMS were also almost all included in the 26 important features of Model 3 of this study, such as gender, coronary artery disease, and medication factors. Moreover, the model performance of Model 3 in this study is much better.

Therefore, overall, the three prediction models of statin effectiveness and safety constructed in this study almost include the important key points during the clinical application of statins, including the effect of LDL reduction, the abnormalities of liver enzymes, and the abnormalities of CK. Not only the model performance is superior, but also the application platform of the model is built, which makes the prediction model of this study more clinically applicable and operable than exploring the gene loci related to statins.

However, it is worth paying attention to the fact that although relevant information mining based on patients’ electronic health records has a better clinical practice application value, it needs to overcome the problems of a single data source, incomplete and unstandardized data, and the existence of unstructured data. Therefore, if the case report form (CRF) can be standardized and designed for the research problem, and the CRF can be embedded into the clinical practice operation link, such as the hospital information system, in conjunction with the information technology personnel. In this way, a large amount of standardized and structured data can be collected in the working scenario, which will greatly improve the efficiency of research and produce more and more valuable research results.

Finally, combining the preliminary ANOVA and the final modeling results, we found that the variables with differences (*p* < 0.05) in the ANOVA contained more information that could be mined. In model 1, of the 29 variables with differences in statistical analysis, 18 variables (62%) were finally used in model construction; in model 2, of the 18 variables with differences in statistical analysis, 12 variables (67%) were finally used in model construction; in model 3, of the 13 variables with differences in statistical analysis, 8 variables (62%) were finally used in model construction. It can be seen that ANOVA can help to identify variables that differ significantly between categories, which are better able to discriminate between categories and thus more helpful in model construction. Therefore, variables with significant differences can improve the generalization performance of the model and improve the classification ability of the model. However, the application of ANOVA relies on some strict assumptions and is less suitable for high dimensional data. In addition, ANOVA is a univariate analysis method, which analyzes the relationship between one feature and the target variable at a time, ignoring the interaction and joint influence between features. To summarize, traditional statistical analysis and machine learning both overlap and have their own focuses, with statistical analysis emphasizing the interpretation and inference of data, and machine learning emphasizing the prediction and application of models. Currently, the two are often used in combination to fully utilize their respective strengths.

This study still has several limitations that need to be acknowledged and addressed. First, the data in this study came from only two healthcare organizations and were exclusively inpatients. This lack of diverse data sources, such as community-sourced data, limits the generalizability of our findings. The study population is not representative of the broader community, which may affect the external validity of the model and cause selection bias. Future research should aim to include a more diverse range of data sources to enhance the robustness and applicability of the model across different settings and populations. Second, as a retrospective study, our research was constrained by the variables available in the existing records. Important factors such as the duration of medication and outpatient follow-up data were not accessible, potentially omitting crucial information that could influence the modeling outcomes. The absence of these variables may lead to an incomplete understanding of the predictors and their relationships, thus introducing information bias. Prospective studies with comprehensive data collection strategies should be conducted to capture these critical variables and provide a more holistic view. Third, missing values were prevalent in our dataset, which could affect the integrity of the information for certain variables. While we applied imputation methods to handle missing data, these approaches might introduce bias or reduce the accuracy of the model. Advanced techniques for handling missing data, such as multiple imputation or the use of robust machine learning algorithms that can better manage missingness, should be explored in future work. Fourth, the study primarily relied on a limited set of data processing and machine learning methods. Exploring a wider array of techniques, including advanced preprocessing methods, feature selection techniques, and different machine learning algorithms, could potentially yield better model performance. Implementing and comparing various approaches can help identify the most effective strategies for building robust predictive models. Finally, continuous validation and constant adjustments are essential to ensure that the model remains relevant and accurate over time. The healthcare environment is dynamic, and the patterns observed in historical data may change. Regular updates to the model with new data, as well as validation in different clinical settings, are necessary to adapt to evolving decision-making needs. In summary, while our study provides valuable insights, addressing these limitations through future research will be critical to improving the model’s validity, reliability, and generalizability. This ongoing effort will help ensure that the model can effectively support clinical decision-making in diverse healthcare scenarios.

## 5 Conclusion

In conclusion, the prediction model for the effectiveness and safety of statins established in this study has good prediction performance (AUC > 0.85), while the construction of the prediction-based platform has good clinical application value to assist clinical decision-making and promote rational drug use. In the future, more in-depth studies are needed to promote the popularization and wider application of the model.

## Data Availability

The original contributions presented in the study are included in the article/Supplementary Material, further inquiries can be directed to the corresponding authors.
