# Supplementary material for: Machine learning-based prediction model for the efficacy and safety of statins
Source: Front Pharmacol. 2024 Jul 29;15:1334929. doi: 10.3389/fphar.2024.1334929 (PMC11317424; doi:10.3389/fphar.2024.1334929)
Supplement: Supplementary file 1 [file DataSheet1.docx]

Supplementary

LDL-C treatment reached the target values in different ASCVD risk groups

| Level of risk | LDL-C[nmol/L(mg/dl)] |
| --- | --- |
| Low/moderate risk | <3.4(130) |
| High risk | <2.6(100) |
| Extremely high risk^#^ | <1.8(70) |

Patients who meet any of the following conditions can be directly classified as high risk or extremely high risk group:

extremely high risk: ASCVD patients

high risk: (1) LDL-C ≥ 4.9mmol/L or TC ≥ 7.2 mmol/L; (2) Patients with diabetes[LDL-C 1.8-4.9mmol/L(or TC 3.1-7.2 mmol/L) and age ≥ 40 years]

The 10-year risk of ASCVD was estimated for those who did not meet the criteria

|  |  | Stratification of SERUM CHOLESTEROL levels (mmol/L) | | |
| --- | --- | --- | --- | --- |
|  | Risk factors* | 3.1≤TC＜4.1 or  1.8≤LDL-C<2.6 | 4.1≤TC＜5.2 or  2.6≤LDL-C<3.4 | 5.2≤TC＜7.2 or  3.4≤LDL-C<4.9 |
| No hypertension | 0-1 | Low risk(<5%) | Low risk(<5%) | Low risk(<5%) |
|  | 2 | Low risk(<5%) | Low risk(<5%) | Moderate risk(5-9%) |
|  | 3 | Low risk(<5%) | Moderate risk(5-9%) | Moderate risk(5-9%) |
| Hypertension | 0 | Low risk(<5%) | Low risk(<5%) | Low risk(<5%) |
|  | 1 | Low risk(<5%) | Moderate risk(5-9%) | Moderate risk(5-9%) |
|  | 2 | Moderate risk(5-9%) | High risk(≥10%) | High risk(≥10%) |
|  | 3 | High risk(≥10%) | High risk(≥10%) | High risk(≥10%) |

If the 10-year risk of ASCVD was moderate and the age ≤ 55 years,

the remaining lifetime risk was evaluated

High ASCVD risk was defined as having any two or more of the following risk factors:

(1) Systolic blood pressure ≥160mmHg or diastolic blood pressure ≥100mmHg;

(2) Non-HDL-C ≥5.2 mmol/L(40mg/dl)

(3) BMI≥28 kg/m^2^

(4) Smoking

# Patients diagnosed with ASCVD (including acute coronary syndrome (ACS), stable coronary heart disease, post-revascularization, ischemic cardiomyopathy, ischemic stroke, transient ischemic attack, peripheral arterial disease) are considered to be extremely high risk.

* Risk factors include smoking, low HDL-C and male ≥ 45 years or female ≥ 55 years; 1mmHg=0.133 kPa

Table S1 The standard for determining whether LDL-C meets the standard

Table S2 The principle for handling the variable of statin dose

|  | Low dose (mg) | Conventional dose(mg) | High dose (mg) |
| --- | --- | --- | --- |
| Atorvastatin | 5 | 10, 20, 30 | 40, 60,80 |
| Rosuvastatin | - | 5, 10, 15 | 20 |
| Pitavastatin | - | 1, 2 | 4 |
| Simvastatin | - | 10, 20 | 40 |
| Fluvastatin | - | 40 | 80 |
| Pravastatin | - | 10, 20 | 40 |
| Amlodipine and Atorvastatin | - | 5/20 | - |

Table S3 ANOVA results for Model 1

|  | meandiff | p-adj | 95% CI | F | PR(>F) | reject |
| --- | --- | --- | --- | --- | --- | --- |
| X1 | -0.0648 | 0.8788 | -0.8976,0.7681 | 0.8611 | 0.3535 | FALSE |
| X2 | 0.0844 | 0 | 0.044,0.1247 | 0.0414 | 0.8388 | TRUE |
| X3 | -1.4139 | 0.0405 | -2.7662,-0.0615 | 3.829 | 0.0505 | TRUE |
| X4 | -0.0555 | 0.0053 | -0.0945,-0.0165 | 2.3228 | 0.1276 | TRUE |
| X5 | -0.062 | 0.0028 | -0.1026,-0.0214 | 0.8457 | 0.3579 | TRUE |
| X6 | -0.6416 | 0.0868 | -1.3761,0.0928 | 1.8036 | 0.1794 | FALSE |
| X7 | 1.1349 | 0 | 0.6038,1.6661 | 0.2114 | 0.6457 | TRUE |
| X8 | 0.0492 | 0.0187 | 0.0082,0.0903 | 2.1283 | 0.1447 | TRUE |
| X9 | -0.0536 | 0.0073 | -0.0927,-0.0145 | 3.2476 | 0.0716 | TRUE |
| X10 | 0.0147 | 0.4646 | -0.0248,0.0542 | 10.6008 | 0.0011 | FALSE |
| X11 | 0.0154 | 0.037 | 0.0009,0.0299 | 5.0241 | 0.0251 | TRUE |
| X12 | 0.0401 | 0.0001 | 0.0201,0.0601 | 0.9063 | 0.3412 | TRUE |
| X13 | 0.0447 | 0.0003 | 0.0208,0.0686 | 2.0772 | 0.1496 | TRUE |
| X14 | 0.0256 | 0.0001 | 0.0125,0.0387 | 7.1012 | 0.0077 | TRUE |
| X15 | 0.0036 | 0.3451 | -0.0039,0.0112 | 1.9397 | 0.1638 | FALSE |
| X16 | 0.0066 | 0.2153 | -0.0038,0.0169 | 0.2798 | 0.5969 | FALSE |
| X17 | 0.0241 | 0.1077 | -0.0053,0.0535 | 0.0585 | 0.8089 | FALSE |
| X18 | 0.0492 | 0 | 0.0289,0.0695 | 5.3683 | 0.0206 | TRUE |
| X19 | 0.0049 | 0.1044 | -0.001,0.0108 | 1.3983 | 0.2371 | FALSE |
| X20 | -0.14 | 0 | -0.1798,-0.1002 | 35.8085 | 0 | TRUE |
| X21 | 0.0003 | 0.9222 | -0.006,0.0066 | 0.296 | 0.5865 | FALSE |
| X22 | 0.0809 | 0 | 0.0559,0.1059 | 18.8506 | 0 | TRUE |
| X23 | 0.5694 | 0.0092 | 0.1408,0.998 | 1.0214 | 0.3123 | TRUE |
| X24 | -0.0164 | 0.0724 | -0.0344,0.0015 | 2.2482 | 0.1339 | FALSE |
| X25 | 0.0303 | 0.0138 | 0.0062,0.0544 | 4.8627 | 0.0275 | TRUE |
| X26 | 0.0016 | 0.904 | -0.0242,0.0273 | 0.6723 | 0.4123 | FALSE |
| X27 | -0.001 | 0.8545 | -0.0115,0.0095 | 1.0388 | 0.3082 | FALSE |
| X28 | -0.0113 | 0.0077 | -0.0196,-0.003 | 2.1729 | 0.1406 | TRUE |
| X29 | -0.002 | 0.5518 | -0.0085,0.0045 | 0.0164 | 0.898 | FALSE |
| X30 | -0.0739 | 0.0002 | -0.113,-0.0348 | 5.5622 | 0.0184 | TRUE |
| X31 | -0.0874 | 0 | -0.1276,-0.0473 | 5.5653 | 0.0184 | TRUE |
| X32 | 0.0063 | 0.3448 | -0.0068,0.0194 | 0.6159 | 0.4326 | FALSE |
| X33 | -0.0072 | 0.0288 | -0.0137,-0.0007 | 0.2864 | 0.5926 | TRUE |
| X34 | -0.0016 | 0.2552 | -0.0043,0.0011 | 2.9146 | 0.0879 | FALSE |
| X35 | 1.9529 | 0.0214 | 0.2894,3.6164 | 0.0268 | 0.87 | TRUE |
| X36 | -0.1174 | 0 | -0.1419,-0.0928 | 90.8152 | 0 | TRUE |
| X37 | -0.3516 | 0 | -0.433,-0.2701 | 66.0533 | 0 | TRUE |
| X38 | -26.9302 | 0.0002 | -41.2957,-12.5646 | 25.9591 | 0 | TRUE |
| X39 | -21.0784 | 0 | -26.6683,-15.4885 | 44.4725 | 0 | TRUE |
| X40 | -0.5971 | 0.164 | -1.4381,0.2439 | 0.0563 | 0.8125 | FALSE |
| X41 | 3.9613 | 0.0295 | 0.3953,7.5272 | 0.9716 | 0.3244 | TRUE |
| X42 | 3.853 | 0.0006 | 1.6536,6.0525 | 5.1395 | 0.0235 | TRUE |
| X43 | 14.3406 | 0 | 8.7553,19.9259 | 7.7073 | 0.0055 | TRUE |
| X44 | -10.2478 | 0.0001 | -15.387,-5.1086 | 0.6722 | 0.4123 | TRUE |

Table S4 ANOVA results for Model 2

|  | meandiff | p-adj | 95% CI | F | PR(>F) | reject |
| --- | --- | --- | --- | --- | --- | --- |
| X1 | -1.1339 | 0.0259 | -2.1314,-0.1363 | 30.9226 | 0 | TRUE |
| X2 | 0.0678 | 0.0057 | 0.0198,0.1157 | 3.9149 | 0.048 | TRUE |
| X3 | 0.6641 | 0.4001 | -0.8832,2.2114 | 0.6321 | 0.4267 | FALSE |
| X4 | 0.0278 | 0.2497 | -0.0196,0.0752 | 0.0576 | 0.8104 | FALSE |
| X5 | 0.0171 | 0.4905 | -0.0316,0.0658 | 0.4563 | 0.4994 | FALSE |
| X6 | 2.6366 | 0 | 1.7737,3.4995 | 17.6671 | 0 | TRUE |
| X7 | 0.3764 | 0.2562 | -0.2735,1.0262 | 0.3662 | 0.5451 | FALSE |
| X8 | -0.0946 | 0.0001 | -0.1432,-0.0459 | 18.7344 | 0 | TRUE |
| X9 | -0.0364 | 0.1232 | -0.0827,0.0099 | 0.2079 | 0.6484 | FALSE |
| X10 | 0.0615 | 0.0104 | 0.0145,0.1086 | 0.0613 | 0.8045 | TRUE |
| X11 | 0.005 | 0.5653 | -0.0119,0.0219 | 0.26 | 0.6102 | FALSE |
| X12 | 0.0335 | 0.0071 | 0.0091,0.0579 | 0.0093 | 0.923 | TRUE |
| X13 | 0.0314 | 0.0319 | 0.0027,0.0601 | 2.2967 | 0.1298 | TRUE |
| X14 | -0.0091 | 0.251 | -0.0247,0.0065 | 2.9295 | 0.0871 | FALSE |
| X15 | 0.0014 | 0.7743 | -0.0079,0.0107 | 0.1363 | 0.712 | FALSE |
| X16 | -0.0429 | 0.0162 | -0.0778,-0.0079 | 7.7582 | 0.0054 | TRUE |
| X17 | 0.071 | 0 | 0.0465,0.0956 | 18.4869 | 0 | TRUE |
| X18 | -0.0016 | 0.6672 | -0.0088,0.0056 | 0.0688 | 0.7932 | FALSE |
| X19 | -0.0207 | 0.3918 | -0.0682,0.0267 | 0.7259 | 0.3943 | FALSE |
| X20 | 0 |  | 0,0 | 0.1785 | 0.6727 | FALSE |
| X21 | -0.0032 | 0.4334 | -0.0112,0.0048 | 1.3734 | 0.2413 | FALSE |
| X22 | 0.0085 | 0.5872 | -0.0223,0.0394 | 2.8315 | 0.0925 | FALSE |
| X23 | 1.7998 | 0 | 1.2913,2.3083 | 26.2078 | 0 | TRUE |
| X24 | -0.0146 | 0.1698 | -0.0355,0.0063 | 2.1269 | 0.1448 | FALSE |
| X25 | -0.0255 | 0.0814 | -0.0542,0.0032 | 1.8258 | 0.1767 | FALSE |
| X26 | 0.023 | 0.1473 | -0.0081,0.0541 | 0.3412 | 0.5592 | FALSE |
| X27 | -0.0006 | 0.9266 | -0.0132,0.012 | 0.4016 | 0.5263 | FALSE |
| X28 | 0 |  | 0,0 | 0.0482 | 0.8262 | FALSE |
| X29 | 0.0059 | 0.2222 | -0.0036,0.0154 | 1.1267 | 0.2886 | FALSE |
| X30 | 0.0079 | 0.036 | 0.0005,0.0153 | 3.1585 | 0.0756 | TRUE |
| X31 | 0.0378 | 0.1153 | -0.0093,0.0849 | 1.0359 | 0.3089 | FALSE |
| X32 | 0.0725 | 0.0028 | 0.025,0.12 | 1.7446 | 0.1867 | TRUE |
| X33 | -0.0001 | 0.9924 | -0.0153,0.0151 | 0.0669 | 0.7959 | FALSE |
| X34 | 0.0038 | 0.2865 | -0.0032,0.0107 | 0.0003 | 0.9873 | FALSE |
| X35 | 0.0013 | 0.4313 | -0.0019,0.0044 | 0.033 | 0.856 | FALSE |
| X36 | 10.13 | 0 | 8.133,12.1269 | 52.56 | 0 | TRUE |
| X37 | -0.0718 | 0.0003 | -0.1111,-0.0325 | 1.3779 | 0.2406 | TRUE |
| X38 | -0.0635 | 0.2227 | -0.1657,0.0386 | 0.065 | 0.7988 | FALSE |
| X39 | -2.636 | 0.7641 | -19.8584,14.5864 | 0.1307 | 0.7178 | FALSE |
| X40 | 2.8549 | 0.4093 | -3.9281,9.638 | 0.1129 | 0.7369 | FALSE |
| X41 | -1.1642 | 0.0209 | -2.1522,-0.1762 | 4.4558 | 0.0349 | TRUE |
| X42 | -1.1549 | 0.7506 | -8.2787,5.9688 | 3.0652 | 0.0801 | FALSE |
| X43 | 2.3316 | 0.465 | -3.9248,8.588 | 1.7001 | 0.1924 | FALSE |
| X44 | -0.1357 | 0.0155 | -0.2456,-0.0258 | 0.5306 | 0.4664 | TRUE |
| X45 | -0.2214 | 0.0046 | -0.3744,-0.0684 | 0.417 | 0.5185 | TRUE |
| X46 | 124.2476 | 0 | 100.141,148.3542 | 77.6001 | 0 | TRUE |

Table S5 ANOVA results for Model 3

|  | meandiff | p-adj | 95%CI | F | PR(>F) | reject |
| --- | --- | --- | --- | --- | --- | --- |
| X1 | -0.3161 | 0.7022 | -1.937,1.3048 | 0.216 | 0.6422 | FALSE |
| X2 | 0.1313 | 0.0013 | 0.0515,0.2112 | 11.7399 | 0.0006 | TRUE |
| X3 | 2.8451 | 0.0226 | 0.4003,5.2899 | 5.8757 | 0.0154 | TRUE |
| X4 | 0.1028 | 0.0084 | 0.0264,0.1791 | 0.0061 | 0.9379 | TRUE |
| X5 | 0.1356 | 0.0009 | 0.0559,0.2154 | 2.7833 | 0.0954 | TRUE |
| X6 | 1.4864 | 0.0544 | -0.0281,3.001 | 0.0005 | 0.983 | FALSE |
| X7 | -0.7058 | 0.1801 | -1.738,0.3264 | 0.8309 | 0.3621 | FALSE |
| X8 | 0.0385 | 0.3512 | -0.0425,0.1196 | 0.7696 | 0.3804 | FALSE |
| X9 | -0.0132 | 0.7388 | -0.091,0.0646 | 0.3749 | 0.5404 | FALSE |
| X10 | -0.0724 | 0.0654 | -0.1493,0.0046 | 1.6697 | 0.1964 | FALSE |
| X11 | 0.0041 | 0.762 | -0.0227,0.031 | 0.5207 | 0.4706 | FALSE |
| X12 | 0.0022 | 0.9006 | -0.0328,0.0373 | 0.0478 | 0.827 | FALSE |
| X13 | -0.0131 | 0.5773 | -0.0594,0.0331 | 0.328 | 0.5669 | FALSE |
| X14 | -0.0032 | 0.7942 | -0.0271,0.0207 | 0.5459 | 0.4601 | FALSE |
| X15 | 0.0249 | 0.0012 | 0.0098,0.0399 | 9.008 | 0.0027 | TRUE |
| X16 | -0.0113 | 0.2892 | -0.0323,0.0096 | 1.1723 | 0.279 | FALSE |
| X17 | 0.0736 | 0.0094 | 0.0181,0.1291 | 2.1592 | 0.1418 | TRUE |
| X18 | -0.0206 | 0.1581 | -0.0492,0.008 | 0.6887 | 0.4067 | FALSE |
| X19 | -0.0042 | 0.416 | -0.0144,0.006 | 0.3719 | 0.542 | FALSE |
| X20 | -0.0577 | 0.1531 | -0.1369,0.0215 | 2.5301 | 0.1118 | FALSE |
| X21 | 0 |  | 0,0 | 0.6875 | 0.4071 | FALSE |
| X22 | 0.0026 | 0.6163 | -0.0076,0.0128 | 0.235 | 0.6279 | FALSE |
| X23 | -0.0357 | 0.1458 | -0.0838,0.0124 | 0.9286 | 0.3353 | FALSE |
| X24 | 0.3277 | 0.4447 | -0.5129,1.1682 | 0.3839 | 0.5356 | FALSE |
| X25 | 0.0034 | 0.8369 | -0.0293,0.0362 | 0.0991 | 0.7529 | FALSE |
| X26 | -0.0225 | 0.3454 | -0.0693,0.0243 | 0.0377 | 0.8461 | FALSE |
| X27 | 0.0069 | 0.7691 | -0.0394,0.0533 | 0.7693 | 0.3805 | FALSE |
| X28 | 0.0014 | 0.8735 | -0.0159,0.0187 | 0.1526 | 0.6961 | FALSE |
| X29 | 0 |  | 0,0 | 1.3996 | 0.2369 | FALSE |
| X30 | 0.0039 | 0.6162 | -0.0115,0.0194 | 0.2402 | 0.6241 | FALSE |
| X31 | -0.0063 | 0.3186 | -0.0188,0.0061 | 1.1548 | 0.2827 | FALSE |
| X32 | 0.0295 | 0.4555 | -0.048,0.1071 | 0.1822 | 0.6695 | FALSE |
| X33 | 0.0097 | 0.8099 | -0.0693,0.0887 | 0.1478 | 0.7007 | FALSE |
| X34 | -0.0262 | 0.0406 | -0.0513,-0.0011 | 3.5909 | 0.0582 | TRUE |
| X35 | 0.0171 | 0.0002 | 0.008,0.0262 | 9.8237 | 0.0017 | TRUE |
| X36 | 0.006 | 0.0101 | 0.0014,0.0105 | 3.2294 | 0.0724 | TRUE |
| X37 | 1.6162 | 0.3425 | -1.722,4.9543 | 0.002 | 0.9648 | FALSE |
| X38 | 0.0044 | 0.88 | -0.053,0.0619 | 0.0107 | 0.9175 | FALSE |
| X39 | -0.0098 | 0.9062 | -0.1724,0.1529 | 0.076 | 0.7828 | FALSE |
| X40 | -10.3698 | 0.4927 | -40.008,19.2684 | 1.4009 | 0.2367 | FALSE |
| X41 | -4.9149 | 0.3882 | -16.0812,6.2514 | 0.3847 | 0.5351 | FALSE |
| X42 | 0.3068 | 0.7177 | -1.357,1.9706 | 0.2465 | 0.6196 | FALSE |
| X43 | 10.4985 | 0 | 6.5657,14.4313 | 18.4229 | 0 | TRUE |
| X44 | 6.5535 | 0.0006 | 2.8102,10.2969 | 3.0361 | 0.0816 | TRUE |
| X45 | 8.6883 | 0.0338 | 0.6661,16.7105 | 1.4113 | 0.2349 | TRUE |
| X46 | 14.2059 | 0.0057 | 4.1419,24.27 | 1.5074 | 0.2196 | TRUE |
| X47 | 0.0507 | 0.5675 | -0.1231,0.2244 | 0.1633 | 0.6862 | FALSE |
| X48 | 0.0565 | 0.6325 | -0.1753,0.2884 | 0.1506 | 0.698 | FALSE |

Table S6 The validation outcome of the 3 models in test set

|  | **Auc** | **Accuracy** | **Precision** | **Recall** | **F1** | **AUPRC** |
| --- | --- | --- | --- | --- | --- | --- |
| Model 1 | | | | | |  |
| Logistic regression | 0.671 | 0.670 | 0.595 | 0.679 | 0.634 | 0.705 |
| Naive bayes | 0.655 | 0.668 | 0.615 | 0.572 | 0.593 | 0.684 |
| Decision tree | 0.783 | 0.784 | 0.729 | 0.779 | 0.753 | 0.800 |
| **Random forest** | **0.924** | **0.919** | **0.868** | **0.954** | **0.909** | **0.920** |
| GBDT | 0.844 | 0.841 | 0.780 | 0.866 | 0.821 | 0.851 |
| Model 2 | | | | | |  |
| Logistic regression | 0.603 | 0.593 | 0.297 | 0.619 | 0.402 | 0.500 |
| Naive bayes | 0.590 | 0.613 | 0.296 | 0.548 | 0.384 | 0.472 |
| Decision tree | 0.671 | 0.797 | 0.548 | 0.445 | 0.491 | 0.558 |
| **Random forest** | **0.922** | **0.944** | **0.857** | **0.884** | **0.875** | **0.888** |
| GBDT | 0.734 | 0.798 | 0.536 | 0.619 | 0.402 | 0.500 |
| Model 3 | | | | | |  |
| Logistic regression | 0.534 | 0.733 | 0.069 | 0.310 | 0.113 | 0.208 |
| Naive bayes | 0.514 | 0.542 | 0.058 | 0.483 | 0.103 | 0.284 |
| Decision tree | 0.719 | 0.837 | 0.184 | 0.586 | 0.281 | 0.397 |
| **Random forest** | **0.929** | **0.988** | **0.925** | **0.862** | **0.892** | **0.897** |
| GBDT | 0.642 | 0.906 | 0.244 | 0.345 | 0.286 | 0.312 |


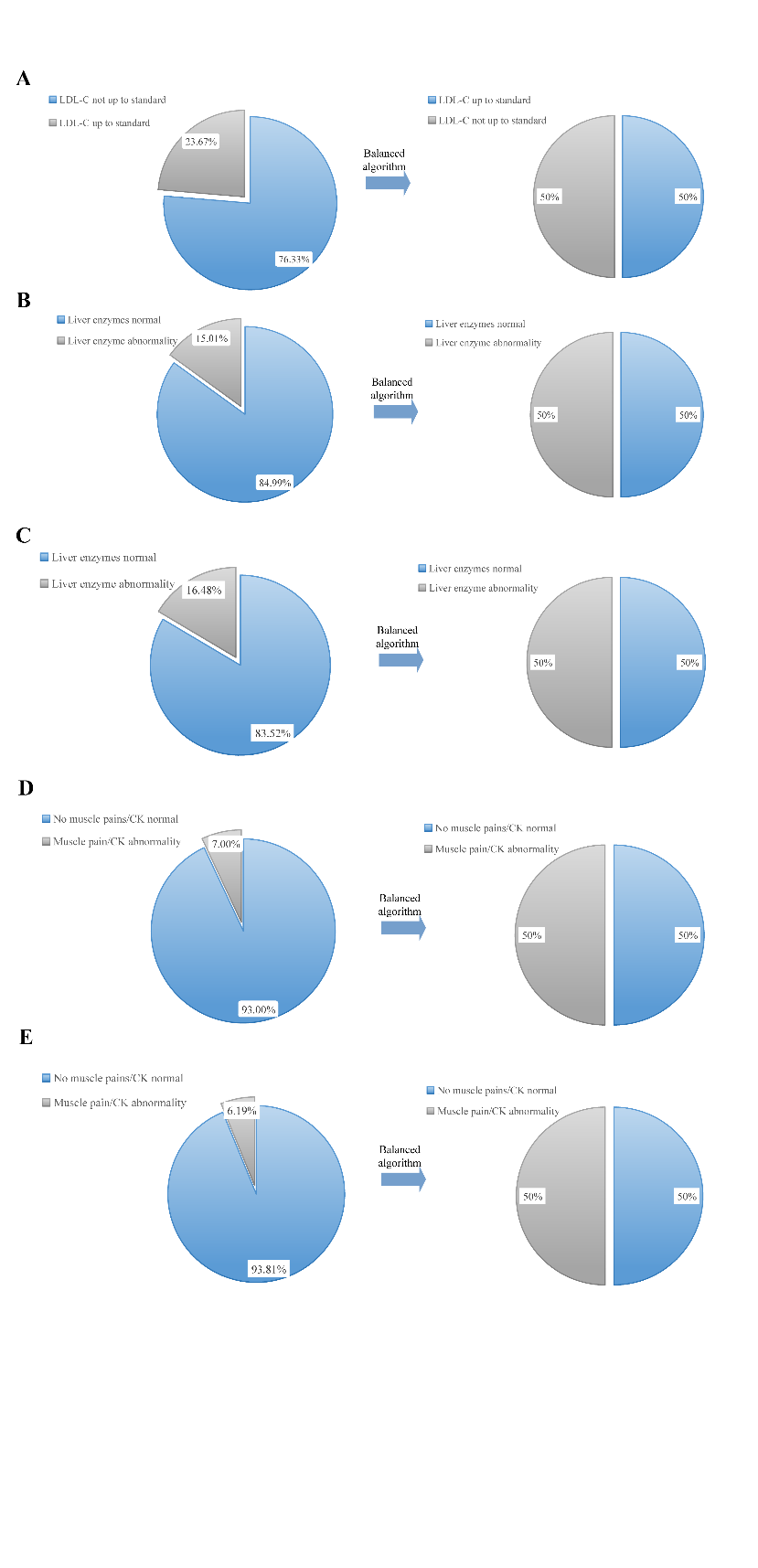


Figure S1 Ratio of positive and negative samples. (A) The distribution of Model 1 with random forest imputation are balanced after using a balanced algorithm. (B) The distribution of Model 2 with no imputation are balanced after using a balanced algorithm. (C) The distribution of Model 2 with random forest imputation are balanced after using a balanced algorithm. (D) The distribution of Model 3 with no imputation are balanced after using a balanced algorithm. (E) The distribution of Model 3 with random forest imputation are balanced after using a balanced algorithm.


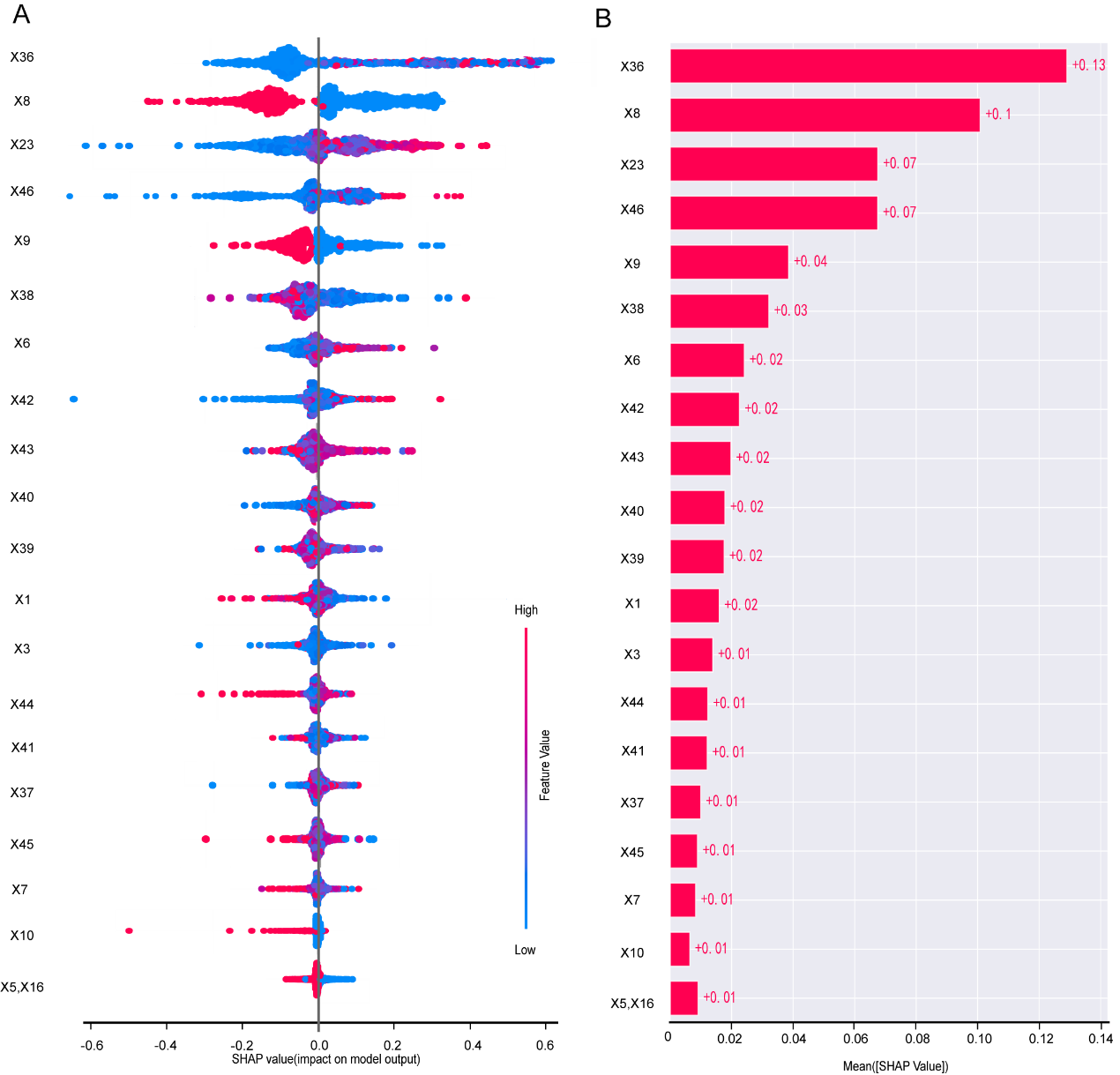


Figure S2 Variable contribution of the Model 2 by SHAP. Summary of SHAP value of each variable(A). Absolute average of SHAP of each variable(B).

X1 age, X3 BMI, X5 drink history, X6 length of stay, X7 number of diseases, X8 T2DM, X9 hypertension, X10 CHD, X16 CKD, X23 number of oral medicines, X36 CRP, X37 HDL, X38 TG, X39 UA, X40 PLT, X41 HCY, X42 Crea, X43 SBP, X44 LDL-C, X45 TC, X46 CK.


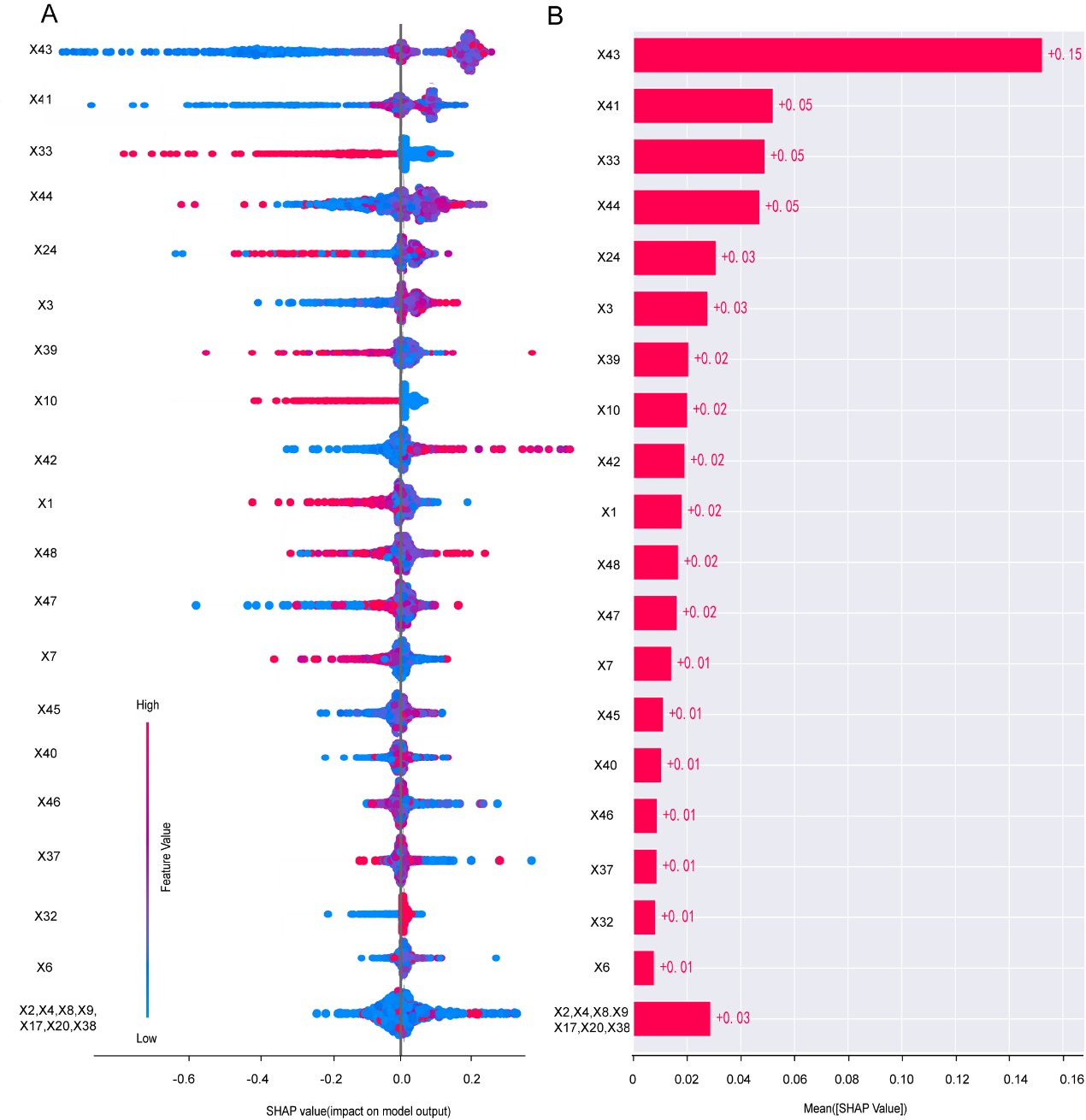


Figure S3 Variable contribution of the Model 3 by SHAP. Summary of SHAP value of each variable(A). Absolute average of SHAP of each variable(B).

X1 age, X2 sex X3 BMI, X4 smoking history, X6 length of stay, X7 number of diseases, X8 T2DM, X9 hypertension, X10 CHD, X17 CKD, X20 cerebral infarction, X24 number of oral medicines, X32 aspirin, X33 clopidogrel, X37 CRP, X38 HDL, X39 TG, X40 UA, X41 PLT, X42 HCY, X43 AST, X44 ALT, X45 Crea, X46 SBP, X47 LDL, X48 TC.

Figure S4 Stain safety prediction platform based on Model 2.

Figure S5 Stain safety prediction platform based on Model 3.
